# Aging and Economic Preferences: Cumulative Meta-Analyses of Age Differences in Risk, Time, Social, and Effort Preferences

**DOI:** 10.1093/geronb/gbad034

**Published:** 2023-03-04

**Authors:** Alexandra Bagaïni, Yunrui Liu, Arzie Bajrami, Gayoung Son, Loreen Tisdall, Rui Mata

**Affiliations:** Faculty of Psychology, Center for Cognitive and Decision Sciences, University of Basel, Basel, Switzerland; Faculty of Psychology, Center for Cognitive and Decision Sciences, University of Basel, Basel, Switzerland; Faculty of Psychology, Center for Cognitive and Decision Sciences, University of Basel, Basel, Switzerland; Faculty of Psychology, Center for Cognitive and Decision Sciences, University of Basel, Basel, Switzerland; Faculty of Psychology, Center for Cognitive and Decision Sciences, University of Basel, Basel, Switzerland; Faculty of Psychology, Center for Cognitive and Decision Sciences, University of Basel, Basel, Switzerland

**Keywords:** Age differences, Cumulative, Economic preferences, Meta-analysis

## Abstract

**Objectives:**

Several theories predict changes in individuals’ economic preferences across the life span. To test these theories and provide a historical overview of this literature, we conducted meta-analyses on age differences in risk, time, social, and effort preferences as assessed by behavioral measures.

**Methods:**

We conducted separate meta-analyses and cumulative meta-analyses on the association between age and risk, time, social, and effort preferences. We also conducted analyses of historical trends in sample sizes and citation patterns for each economic preference.

**Results:**

The meta-analyses identified overall no significant effects of age for risk (*r* = −0.02, 95% CI [−0.06, 0.02], *n* = 39,832) and effort preferences (*r* = 0.24, 95% CI [−0.05, 0.52], *n* = 571), but significant effects of age for time (*r* = −0.04, 95% CI [−0.07, −0.01], *n* = 115,496) and social preferences (*r* = 0.11, 95% CI [0.01, 0.21], *n* = 2,997), suggesting increased patience and altruism with age, respectively. Equivalence tests, which compare these effects to practically important ones (i.e., *r* = |0.1|), however, suggest that all effects are of trivial significance. The analyses of temporal trends suggest that the magnitude of effects and sample sizes have not changed significantly over time, nor do they dramatically affect the extent that articles are cited.

**Discussion:**

Overall, our results contrast with theories of aging that propose general age effects for risk and effort preferences, yet provide some but tenuous support for those suggesting age-related changes in time and social preferences. We discuss implications for theory development as well as future empirical work on economic preferences.

Aging is thought to be associated with changes in decision-making that can carry long-term consequences for oneself as well as others, including choices about financial investment, savings, donations, or effort expenditure. Economic preferences reflect how individuals tend to make associated trade-offs about risk, time, social, or effort dimensions when making such choices and there has been considerable interest in understanding how and to what extent such preferences change with age (e.g., Best & Charness, 2015; Seaman et al., 2022; Sparrow et al., 2021; Westbrook et al., 2013). The empirical results concerning economic preferences have, however, been mixed, and there have been recent calls to examine the research practices associated with aging research and harmonize both theories and methods to advance the study of age differences in economic preferences (e.g., Frey et al., 2021). For example, some researchers have voiced concern about a potential tendency to exclusively report significant age differences in the aging literature (e.g., Isaacowitz, 2018, 2020) or how certain stylized facts about the link between aging and economic preferences may reflect the work of a few seminal studies that are based on relatively small sample sizes and are not representative of the literature as a whole (e.g., Seaman et al., 2022).

In this work, we aim to contribute to integrating both theory and empirical knowledge about age differences in economic preferences by providing a comprehensive research synthesis of this literature to assess how the different existing theories in this domain match with the empirical evidence accumulated over time. Taking stock of the amount and time course of how evidence accumulates over time can provide insights into the history of the field, the impact of evolving research practices (e.g., study designs, sample sizes, statistical approaches), and the stability of the knowledge acquired (Koricheva et al., 2013; Kulinskaya & Mah, 2022). We thus aim to provide an overall assessment of how different theories of aging are supported or rejected by current empirical evidence and provide input for both theory development and future empirical work in the domain of economic preferences.

## Economic Preferences: Risk, Time, Social, and Effort

In this study, we focus on age-related differences in four domains of economic preference: risk, time, social, and effort-related preferences. Table 1 provides a summary of these constructs along with examples of tasks commonly used in the psychological literature to assess them. Risk preference can be defined as the propensity of an individual to prefer options offering varying (monetary) rewards over certain ones. Popular tasks involve choosing between gambles of varying levels of (learned or described) rewards or probabilities (e.g., Holt & Laury, 2002). Time preference is defined by how much an individual discounts the value of future rewards over sooner ones. Most often a discounting rate is estimated based on the choices an individual makes between immediate rewards and larger delayed rewards in a temporal discounting task (Frederick et al., 2002). Social preference reflects an individual’s inclination to forgo resources for oneself for the sake of another individual. The dictator game (Forsythe et al., 1994) is a commonly used task where a player chooses to donate a certain amount of real or hypothetical money to an undisclosed participant. Lastly, effort preferences are typically conceptualized as effort discounting and calculated by how much the subjective value of a reward decreases as a function of the cognitive or physical effort needed to acquire it (e.g., Ostaszewski et al., 2013).

**Table 1.** Description of Economic Preferences

Preference	Description	Trade-off	Examples of measures
Risk	Propensity to choose options with higher variance in potential monetary outcomes	Smaller-sure vs larger-uncertain	Cups Task, Lotteries, Bal- loon Analogue Risk Task
Time	Valuation placed on receiving a monetary reward sooner compared to later	Smaller-sooner vs larger-later	Temporal Discounting Task
Social	Propensity of an individual to engage in behavior motivated by the concern for others and for the sake of others	Smaller-self vs larger-other	Dictator Game, Social Dis counting Task
Effort	Valuation placed on a monetary reward after exerting physical or cognitive effort.	Low reward-low effort vs large reward-large effort	Effort Discounting Task

## Theoretical Accounts Predicting Age Differences in Economic Preferences

As outlined earlier, a number of theoretical approaches have made predictions about the life-span development of the economic preferences. In what follows, we discuss a number of such theories with a particular focus on those that have been used to make predictions across different types of economic preferences.

Socioemotional selectivity theory (cf. Carstensen, 2006) is a prominent motivational theory that has been used to derive predictions across a number of economic preferences, including risk, time, and social preferences. It postulates that with age, an individual’s future time horizon shrinks, which results in a shift in goal orientation, from future to present oriented as well as from the self to others. There has been some discussion about the empirical status of socioemotional selectivity theory and how it can be distinguished from other motivational theories (cf. Depping & Freund, 2011) but there seems to be some consensus that the theory predicts a decrease in risk taking, increased temporal discounting, as well as increased altruism with age (cf. Frey et al., 2021; Seaman et al., 2022; Sparrow et al., 2021).

Other theories have proposed that age differences in economic decisions can be the result of relatively general neurological changes. For example, the dopaminergic neuromodulation hypothesis posits that a decline in dopaminergic functioning reduces older adults’ responses toward rewards. Therefore, older adults in comparison to younger adults are less motivated to obtain rewards, leading to a reduction in the propensity to take risks or exert effort to obtain a larger reward, as well as a decreased need to obtain an immediate reward (cf. Frey et al., 2021; Seaman et al., 2022; Westbrook et al., 2013).

Other theories consider the interaction between age-related cognitive decline and task characteristics. Specifically, the confound hypothesis suggests that there may be differences between types of tasks as a function of their cognitive demands and different aspects of cognitive functioning (e.g., fluid vs crystallized aspects) that can moderate age effects (cf. Mata et al., 2011). This is particularly applicable to risk and time preference tasks, in which researchers have shown that estimates for risk preference and temporal discounting can appear to increase or decrease as a function of task demands or analytic confounds (cf. Frey et al., 2015; Olschewski et al., 2018).

Other theories have focused on other nonpsychological causes that co-vary with age, including socioeconomic factors, such as one’s social network and financial wealth, that can shape individuals’ economic preferences. For example, the accumulation of social and economic capital implies reduced striving for such resources across the life span, leading to changes in financial risk taking and social behavior (cf. Frey et al., 2021; Mayr & Freund, 2020).

Aside from theories that make predictions across several economic preferences, a number of preference-specific theories have also been advanced. For example, for risk preference, evolutionary signaling theory presents risk taking as an indication of fitness that is most relevant to younger adults that need to signal fitness for reproductive reasons (cf. Frey et al., 2021). For time preference, some have suggested that the perception of time changes, whereby with age, the impression that time goes by more quickly becomes more common, which can reduce the perception of amount of time to wait to obtain a larger reward, and in turn increases one’s willingness to wait (cf. Seaman et al., 2022). Concerning prosocial behavior, the intuitive-prosociality hypothesis describes altruism as an intuitive response that tends to increase with age (cf. Mayr & Freund, 2020). Lastly, regarding effort, selective engagement theory postulates that age-related increases in the perception of costs related to a task decrease the willingness to expend effort (cf. Hess et al., 2021).

All in all, this short survey highlights the rather heterogeneous character of theories, spanning motivational, cognitive, and ecological factors, and the plethora of mechanisms proposed in the past literature. Table 2 summarizes these and other theoretical accounts and lists relevant references to provide an overview of predictions about how age is associated with each of the four types of economic preferences. In this paper, we examine the match between these predictions and the empirical evidence across types of preferences in a systematic fashion. Such integrative efforts are important as they provide an assessment of the scope of theories and help us gain a better sense of their strengths and limitations. Additionally, examining a theory across multiple domains can help identify inconsistencies or gaps, and provide insight into how the theory can be refined or expanded.

**Table 2.** Theoretical Accounts of Associations Between Age and Economic Preferences

Theoretical account	Preference			
	Risk	Time	Social	Effort
Affective forecasting (cf. Seaman et al., 2022)	.	D	.	.
Cognitive control hypothesis (cf. Frey et al., 2021)	D	.	.	.
Confound hypothesis (cf. Frey et al., 2021; Mata et al., 2011; Seaman et al., 2022)	I/D	I/D	.	.
Dopaminergic neuromodulation hypothesis (cf. Frey et al., 2021; Seaman et al., 2022; Westbrook et al., 2013)	D	D	.	I
Intuitive-prosociality hypothesis (cf. Mayr & Freund, 2020)	.	.	I	.
Evolutionary signaling hypothesis (cf. Frey et al., 2021)	D	.	.	.
Future self-continuity hypothesis (cf. Löckenhoff, & Rutt, 2017)	.	D	.	.
Mortality (cf. Seaman et al., 2022)	.	U	.	.
Motivational reorientation theory (c.f. Frey et al., 2021; Hess et al., 2021)	I/D	.	.	I/D
Psychosocial development theory (cf. Mayr & Freund, 2020)	.	.	∩	.
Resource accumulation (c.f. Frey et al., 2021; Mayr & Freund, 2020)	D	.	I	.
Selective engagement theory (cf. Hess et al., 2021)	.	.	.	I
Self-efficacy theory (cf. Hess et al., 2021)	.	.	.	I/D
Social-investment theory (cf. Frey et al., 2021)	D	.	.	.
Socioemotional selectivity theory (cf. Frey et al., 2021; Seaman et al., 2022; Sparrow et al., 2021)	D	I	I	.
Time perception (cf. Seaman et al., 2022)	.	D	.	.

*Notes*: I: Increased risk taking/temporal discounting/altruism/effort discounting with age. D: Decreased risk taking/ temporal discounting/altruism/effort discounting age. U: U-shaped relation between risk taking/temporal discounting/altruism/effort discounting and age. ∩: Inverse U-shaped relation between risk taking/temporal discounting/altruism/effort discounting and age. Dot: Not applicable.

*Source*: Adapted from Frey et al. (2021) and Seaman et al. (2022).

## Past Empirical Evidence

Given the diversity of theoretical approaches in place, it may not be surprising that existing reviews and meta-analyses on the effect of age on risk, time, or social preferences report findings that are not fully consistent with all the proposed theories (Best & Charness, 2015; Mata et al., 2011; Seaman et al., 2022; Sparrow et al., 2021). In the context of age-related differences in risk preference, the most recent meta-analysis of behavioral measures found no overall effect of age on risk preference but reported that age differences depend on their context (health vs monetary) and domain, specifically, gains versus losses (Best & Charness, 2015). An earlier meta-analysis also found no overall effect of age on risk preference but did report suggestive evidence that age differences may be evident for tasks that involve learning from experience (Mata et al., 2011). For time preference, a recent meta-analysis reported no significant main effect of age (Seaman et al., 2022). In line with theory, a recent meta-analysis that synthesized evidence on age-related differences in social preference involving a mix of measure types (behavioral tasks, self-reports) reported a medium-sized effect of age, with older adults showing greater altruistic tendencies than younger adults (Sparrow et al., 2021). Finally, thus far, no meta-analysis has been conducted on age differences in effort discounting, but primary studies show conflicting results regarding age differences (e.g., Hess et al., 2021; Seaman et al., 2016).

Despite the past empirical work including research synthesis in this area, it is still difficult to adequately compare the empirical results to theories for several reasons. First, each meta-analysis captured the state of the literature at a specific point in time and thus may have captured different amounts and types of evidence that bear on the theories in question. Second, the meta-analyses did not share the same eligibility criteria, such as sample characteristics, study designs, or types of measures (behavior vs self-report). Third, more broadly, past syntheses have not assessed how evidence on age effects accumulated over time and to what extent changing research practices such as the introduction of specific paradigms or study characteristics (sample size and study context) have influenced the estimates of age differences in economic preferences or their impact. We believe, however, that putting our estimates of age effects in a historical context could be important to either assuage or strengthen concerns about the status of the aging literature (e.g., Isaacowitz, 2020).

## Overview of the Current Study

In this study, we aim to address the limitations of past work by offering an updated overview of age effects on risk, time, social, and effort preferences. We focus specifically on studies that have investigated age differences in economic preferences as measured through behavioral tasks involving financial decisions. The main rationale for focusing on behavioral measures in the financial domain is to maximize comparability across types of economic preferences. This is important because recent work suggests that different measure types (behavioral measures vs self-reports) do not always produce similar results concerning age effects on economic preferences (e.g., Frey et al., 2021). Consequently, in our work, we update and harmonize previous meta-analyses by focusing specifically on behavioral tasks in the financial domain. Relatedly, this also allows us to explore the role of a large range of theoretically and empirically motivated moderators across all preferences (see Supplementary Table 1 for an overview). Further, we extend past syntheses by conducting cumulative meta-analyses to gain insight into how estimates of age effects changed over time as evidence accumulated in the literature. A cumulative meta-analysis is the process of updating meta-analytic results by incorporating new evidence (Lau et al., 1992), and this approach can help detect historical trends, evaluate evidence sufficiency, and possibly identify selective reporting, such as time-lag bias or the Proteus phenomenon (i.e., the tendency for early replications of a scientific work to contradict the original findings; Ioannidis & Trikalinos, 2005; Koricheva et al., 2013; Young et al., 2008), which has been implied in past aging work (Seaman et al., 2022). Lastly, some areas of psychology have seen noticeable changes over time that are linked to new research practices (e.g., conducting online studies) that allow for the convenient sampling of larger samples and can have consequences for the quantity and quality of data (Sassenberg & Ditrich, 2019). Consequently, we explore the link between time of publication and sample sizes as a way to assess whether research practices have changed over time in the context of economic preferences, as well as assess studies’ impact by analyzing their historical citation patterns. Overall, we hope to determine the robustness and stability of estimates of age effects on economic preferences so as to be able to draw robust conclusions about the match between the observed empirical patterns and extant theoretical predictions.

## Method

Our research synthesis approach involved two steps. First, we conducted a scoping review of the aging literature to identify existing meta-analyses that have estimated age differences in economic preferences (see the Supplementary Appendix for details on our search strategy and results). Our main goal was to make sure we included all eligible primary studies from these existing reviews. Second, we performed a search for additional primary studies following the Preferred Reporting Items for Systematic Reviews and Meta-Analyses (PRISMA) guidelines (Page et al., 2021) with the goal of complementing the coverage of past research syntheses. Subsequently, we describe the steps involved in the search, screening, and data extraction for primary studies on age differences in risk, time, social, and effort preferences.

### Literature Search

For time preference, we complemented the list of primary studies from Seaman et al.’s (2022) meta-analysis with more recent studies, whereas for risk, social, and effort preferences, we conducted whole new searches (for papers published until November 1, 2022; Supplementary Table 2). We did not complement previous meta-analyses (Best & Charness, 2015; Mata et al., 2011; Sparrow et al., 2021) due to significant differences with the eligibility criteria, analysis, and coding used by Seaman et al. (2022).

The searches returned 2052, 315, 460, and 510 candidate studies to screen for risk, time, social, and effort preferences, respectively.

### Screening

To screen the articles resulting from the search, we devised a set of criteria that we harmonized and applied across all four preferences. We used the same criteria as Seaman et al. (2022), with the exception that we excluded unpublished studies (that would be difficult to place in a historical analysis) and studies that collected data while participants underwent brain imaging, brain stimulation, or pharmacological studies (that would decrease comparability). An overview of the general and preference-specific criteria is available in Supplementary Table 3.

From the search results, we first screened studies based on the title and abstract, and removed 1817, 232, 414, and 441 studies for risk, time, social, and effort preference, respectively. Individual study members then reviewed the remaining full-text articles. We observed that certain articles that we included for the analysis investigated multiple economic preferences. Therefore, we complemented the list of included articles across preferences by adding articles that had been included in the meta-analysis of one economic preference and met the criteria of another but that had not been identified in the search of this one. In the end, a total of 57, 50, 13, and 6 published articles were included in the analysis of age differences in risk, time, social, and effort preference, respectively. The process is illustrated in separate PRISMA flow diagrams (Supplementary Figures 2–5).

### Extraction and Effect Size Calculation

Once studies had been selected for inclusion, we extracted the information necessary for the analysis. For data extraction, two individual study members extracted the data from each study to ensure the accuracy of the extracted information. We extracted information either directly from the articles, from figures using the metaDigitise package in R (Pick et al., 2018), or when available, the raw study data. Studies that provided either insufficient information or overpopulated figures from which it was not possible to extract reasonably accurate outcome values or approximate sample sizes were excluded from the analyses.

Because the included studies quantified the association between age and economic preferences using different metrics and study designs, before combining all the outcomes in the meta-analysis, we first converted these into correlation coefficients.

For studies using an extreme group design where the outcome variable was continuous but age was dichotomous (i.e., younger and older adults), we converted the standardized mean difference (or *t* test value) between two age groups into a point-biserial correlation coefficient. However, if Pearson’s *r* correlation coefficients between age and outcomes had been or could be calculated, these were selected for the analysis. For designs where both age and the outcome variable were measured continuously, we used Pearson’s *r* correlation coefficient.

We coded all effect sizes such that higher values indicated either increasing risk taking, altruism, temporal discounting, or effort discounting with age. For extreme group designs, we focused on the differences between the youngest and oldest adult samples, and did not include in the analyses differences with intermediate age groups.

By following this procedure, we created four sets of effect sizes (i.e., one for each preference), which resulted in a total of 369 effect sizes with data from 141,794 individuals. In addition, to subsequently assess the effect of certain moderators on the individual effect sizes, we coded (a) the type of study design from which it originated (i.e., extreme design or continuous), (b) the effect size metric (i.e., Pearson’s *r* or point-biserial correlation), (c) whether the task involved hypothetical or incentivized decisions, (d) decisions from experience or description, (e) whether these decisions were made in the gain or loss domain, (f) study context (i.e., online or in person), and (g) proportion of females in the sample (see Supplementary Table 1 for rationale). In addition, we calculated the age range of the sample. For Pearson’s *r* correlations, we computed the age difference (in decades) between the youngest and oldest participants, and for point-biserial correlations, the difference between the mean age (in decades) of the oldest and youngest adult groups. If this information was missing, we used the midpoint of the age range of each group (e.g., if participants in a group were between 18 and 30 years of age, we used 24 as the value).

A detailed overview of the included studies, as well as the data and code used to compute effect sizes, is available in the online repository.

### Analysis

We used the R statistical environment (R Core Team, 2020) and the metafor package (Viechtbauer, 2010) to perform the analyses. The analysis code is available in the online repository.

#### Meta-analysis

Some studies reported multiple outcomes (e.g., multiple conditions, multiple behavioral indices); instead of selecting one outcome per study or aggregating these, we entered all outcomes in the meta-analytic. For each data set, we fitted a three-level meta-analysis model using restricted maximum likelihood (REML) estimation. The model included random effects at the estimate (i.e., Level 2 cluster variable) and study (i.e., Level 3 cluster variable) levels, and accounted for the dependence of effect sizes by allowing the sampling errors within studies to be correlated. A correlation of 0 would indicate that the outcomes are independent whereas a correlation of 1 would indicate full correspondence; for our analyses, we opted for a correlation of 0.5. To explore whether the level of correlation between the outcomes of the same study had an influence on the results, we ran sensitivity analyses with correlations varying between 0.1 and 0.9 (Supplementary Figure 12). Additionally, we applied robust variance estimation methods to obtain more precise model estimates (Pustejovsky & Tipton, 2022).

In addition to assessing the statistical significance (alpha = 5%) of the meta-analytic effect size estimates, we assessed their practical significance by performing equivalence tests (Lakens, 2017; Lakens et al., 2018). Based on standard guidelines (Cohen, 1988), we chose *r* = |0.1| as the smallest effect size of interest; this is defined as a small effect, but representative of the correlations found in individual differences research (Bosco et al., 2015; Gignac & Szodorai, 2016).

To identify whether any study was particularly influential, we conducted on each set of effect sizes an influence analysis by computing the pooled effect size omitting one study at a time (Supplementary Figure 11).

Lastly, informed by previous meta-analyses and theory, we estimated a series of meta-regression models to test whether some of the heterogeneity in age-related effects could be explained by certain moderators (see Supplementary Table 1 for an overview).

#### Cumulative meta-analysis and historical trends

Cumulative meta-analyses can be conducted by adding effect sizes to the meta-analytic model in chronological order by study or by publication year. With the latter approach, we can better examine temporal trends while also accounting for cases when more than one study can be published within the same year, which, depending on how they are entered in the cumulative meta-analysis, could affect the shape of the plots (Koricheva et al., 2013; Leimu & Koricheva, 2004). Therefore, we prioritize reporting the results from repetitively fitting the above-specified three-level meta-analysis model by adding the effect sizes by publication year. The results of the cumulative meta-analyses conducted at the study level are reported in Supplementary Appendix.

To explore historical trends in effect sizes, we included in a meta-regression model the number of decades the study had been published as of 2022 as a moderator. Additionally, we explored changes in sample sizes (log) over time by fitting a linear regression. Further, considering the predictions made by certain theories (e.g., socioemotional selectivity theory, confound hypothesis), for risk preference, we explored the accumulation of evidence and temporal trends for each domain (i.e., gain, loss, and mixed) and task type (i.e., description and experience) separately. This amounts to conducting an independent estimation of residual between-studies variance for the two moderators (cf., Rubio-Aparicio et al., 2020). Lastly, we explored the relation between yearly citations with effect sizes and sample sizes. Details on the method used and results are available in the Supplementary Appendix.

#### Publication bias

For all four sets of effect sizes, we performed various analyses, including Egger’s tests and *p*-curve tests, and produced funnel plots to check for publication bias in the published literature (see Supplementary Appendix for details on our approach and results).

## Results

There were differences across the four economic preferences in the number of effect sizes, their distribution, and study sample size (Supplementary Figure 6). Here, we report on the overall effect of age based on cluster-robust inference and the accumulation of evidence for each economic preference separately by displaying effect sizes by year of publication (study-level estimates are included in Supplementary Appendix and Supplementary Figure 7).

### Risk

#### Meta-analysis

The meta-analysis of the relevant 193 effect sizes suggests age is not associated with risk preference (*r* = −0.02, 95% CI [−0.06, 0.02], *p* = .251). Equivalence tests showed that the effect fell within the equivalence bounds (*z* = 3.73, *p* < .001; Figure 2A).

**Figure 1.**
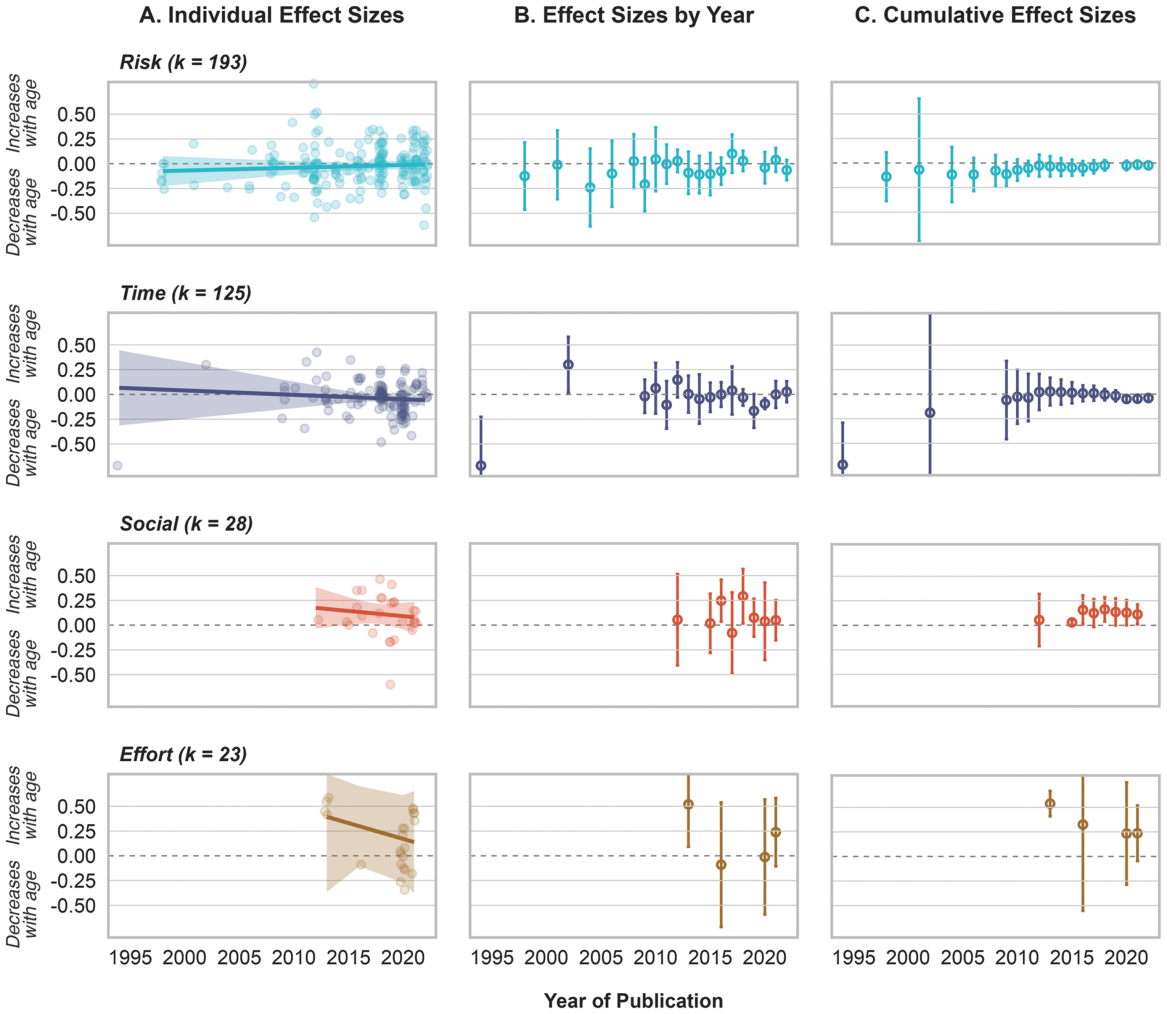
Meta-analytic results of the effect of age on risk (*k* = 193, *s* = 62, *n* = 39832), time (*k* = 125, *s* = 54, *n* = 115496), social (*k* = 28, *s* = 15, *n* = 2997), and effort (*k* = 23, *s* = 7, *n* = 571) preferences. (A) Scatter plots of the individual effect sizes plotted as a function of the publication year with model predictions and 95% CI. (B) Aggregated forest plots of the three-level meta-analytic model with effect sizes pooled by year with 95% CI. (C) Forest plots of the cumulative effect sizes and 95% CI based on cluster-robust inference. CI = confidence interval.

**Figure 2.**
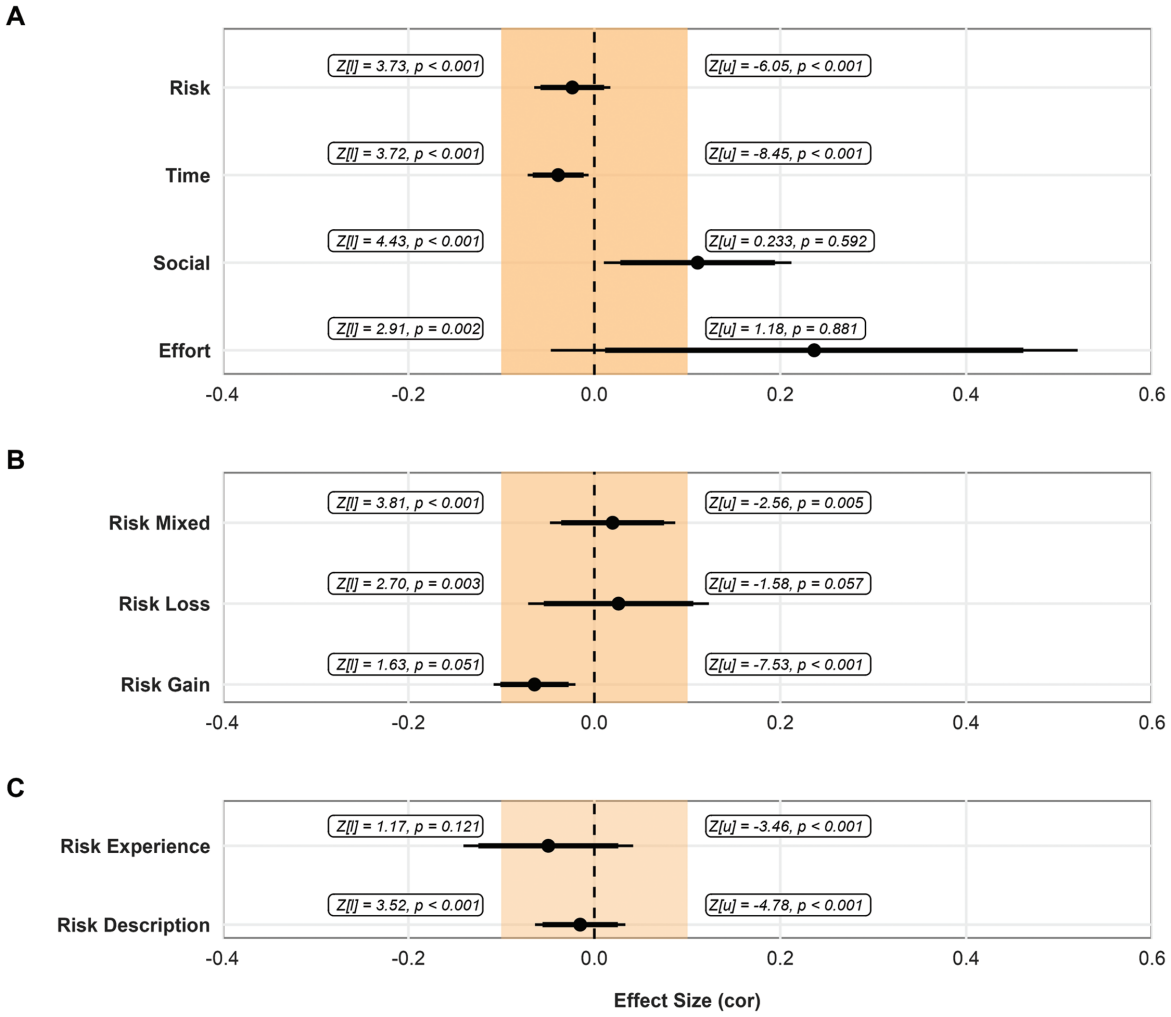
Equivalence test results (against the [u]pper and [l]ower equivalence bounds) for the estimated pooled effect sizes (dots), with 95% (thin lines) and 90% (thick lines) cluster-robust confidence intervals. The shaded section represents the equivalence bounds (*r* = |0.1|). (A) Pooled effect size estimates from the separate three-level meta-analysis models for age differences in risk (*k* = 193), time (*k* = 125), social (*k* = 28), and effort (*k* = 23) preference. (B) Pooled effect size estimates from the separate three-level meta-analysis models for age differences in risk taking in the gain (*k* = 106), loss (*k* = 46), and mixed (*k* = 41) domain. (C) Pooled effect size estimates from the separate three-level meta-analysis models for age differences in risk taking in decisions from description (*k* = 147) and experience (*k* = 44).

To investigate effect size heterogeneity, *Q*(*df* = 192) = 2,232.19, *p* < .001, we estimated in separate three-level meta-regressions the moderating role of (a) sample age range, (b) gender, (c) effect size metric, (d) study design, (e) incentivization, (f) domain, (g) task type, and (h) study context. We find a small but significant decrease in risk taking with age in the gain domain, none of the other moderators were statistically significant (Supplementary Table 4). Further, when we performed separate analyses for each domain (Supplementary Figure 8), allowing the amount of residual heterogeneity to differ between domains (vs using a pooled estimate as with the meta-regression), this effect remained (*r* = −0.06, 95% CI [−0.11, −0.02], *p* = .005), with an equivalence test showing that this effect fell outside the equivalence bounds (Figure 2B). Further, for decisions from experience, although in the separate analyses the effect remained statistically nonsignificant, we cannot reject that the association between risk taking in these tasks and age is at least −0.1 (Figure 2C).

#### Cumulative meta-analysis and historical trends

From Figure 1C, we observe that there was never any evidence supporting age differences in overall task-based risk taking. Since 2010, effect sizes have remained relatively stable, and oscillated between −0.07 and −0.01. Further, when splitting effect sizes by domain or task, as shown in Supplementary Figures 8C and 9C, the effect of age is not particularly stable over time and the number of effect sizes in each category is quite heterogeneous, thus warranting additional evidence in each domain and type of task. There was no linear relation between effect size (*b* = −0.03, *p* = .434), sample size, and number of decades the paper had been published for (Supplementary Figure 10). Further, we find a statistically significant linear effect of sample size on citations but not of effect size (Supplementary Figure 15, Supplementary Tables 10 and 11).

#### Summary

To summarize, we find overall no effect of age on risk preference. Concerning moderators, and contrary to previous syntheses, we find no strong support for the idea that age differences vary systematically as a function of the learning and memory demands of the task as captured through the distinction between description versus experience (Mata et al., 2011). However, we find a small negative effect of age in the gain domain in line with past meta-analytic work (Best & Charness, 2015) and some theoretical predictions (Depping & Freund, 2011). Concerning historical trends, we find no evidence of trends in effect sizes, sample sizes, or citations. Finally, we find overall no evidence of publication bias (see Supplementary Appendix).

### Time

#### Meta-analysis

The meta-analysis of the 125 effect sizes shows a small negative effect of age on time preference (*r* = −0.04, 95% CI [−0.07, −0.01], *p* = .020). However, equivalence tests showed that the effect fell within the equivalence bounds (Figure 2A).

To understand the possible differences between the individual effect sizes (*Q*[*df* = 124] = 496.69, *p* < .001), we conducted separate meta-regressions to investigate the moderating role of (a) sample age range, (b) gender, (c) effect size metric, (d) study design, (e) incentivization, and (f) study context. We noted a significant difference in effects for study context: We find an age-related effect for temporal discounting in online studies (*b* = −0.05, 95% CI [−0.08, −0.02], *p* = .002). Further, we also note the effects of study design and effect size metric (Supplementary Table 5). Lastly, there is also a difference due to incentives, but given that close to 90% of the studies included in our analyses involve hypothetical payments, we treat this difference with caution.

#### Cumulative meta-analysis and historical trends

Figure 1C shows that the first study published in 1994 (i.e., Green et al., 1994) found a large age difference, with older adults exhibiting less temporal discounting than younger adults (*r* = −0.72, standard error [*SE*] = 0.22). However, in 2002, the second study was published (i.e., Kirby et al., 2002) reporting evidence in the opposite direction (*r* = 0.30, *SE* = 0.07), and when combining this with the evidence from the first study, it led the pooled effect size to shift closer to zero, increased the uncertainty around it, and made it statistically nonsignificant (*r* = −0.19, 95% CI [−6.66, 6.28], *p* = .774). Since then, age differences in temporal discounting have remained nonsignificant, with pooled effect sizes nearing zero, but more recently, such small negative effects reached statistical significance. We tested for the presence of the Proteus phenomenon, which is when a large and extreme result is first published but is followed by the publication of less extreme results and can be indicative of publication bias (Ioannidis & Trikalinos, 2005; Young et al., 2008). We followed the approach by Koricheva et al. (2013) and compared the effect size and variance of the first study with the mean effect size and variance of the rest of the published studies. We obtained a *z* value of 2.71, *p* = .007, suggesting that the study by Green et al. (1994) differed significantly above chance from the other results.

The historical analyses showed no linear effect of decades since the paper has been published on the size of the effects (*b* = 0.04, *p* = .538). Furthermore, there was no statistically significant linear relation between publication year and sample size (Supplementary Figure 10). Concerning the citation analyses, we do not detect any discernible trend (Supplementary Figure 15).

#### Summary

We observe a small negative effect of age on time preference; however, equivalence tests show that this effect can be considered trivially small. Regarding historical trends, we find no evidence of trends in effect sizes, sample sizes, or citation patterns. Concerning publication bias, we find evidence of a Proteus phenomenon but no other evidence of bias (see Supplementary Appendix).

### Social

#### Meta-analysis

The meta-analysis of the 28 effect sizes revealed a small positive effect of age on social preference, suggesting that altruistic behavior as measured by behavioral tasks increases with age (*r* = 0.11, 95% CI [0.01, 0.21], *p* = .033). This is consistent with the results from the recent meta-analysis by Sparrow et al. (2021), who also reported a positive, albeit larger, effect size (*r* = 0.24, 95% CI [0.12, 0.35], *p* = .001; see Author Note^1^). Further, this effect also falls outside the equivalence bound, but is not distinguishable from the upper bound (Figure 2A).

As there was considerable heterogeneity in the effect sizes (*Q*[*df* = 27] = 265.65, *p* < .001), we also explored the potential moderating role of (a) sample age range, (b) gender, (c) effect size metric, (d) study design, (e) incentivization, and (f) study context. We find that this positive age effect is mainly driven by point-biserial correlation coefficients (*k* = 14; *b* = 0.17, 95% CI [0.05, 0.29], *p* = .011). Out of the rest of the moderators, we also noted an effect of the study design (Supplementary Table 6).

#### Cumulative meta-analysis and historical trends

Relative to age differences in risk or time preference, age differences in social preference have been more recently investigated (Figure 1). Initially, no significant age differences were reported; however, with additional studies reporting larger (and statistically significant) effect size estimates, the cumulative estimate began to shift away from zero in the positive direction. It reached a peak (*r* = 0.16, *SE* = 0.05) in the year 2018 (includes 10 studies and 12 effect sizes); however, since then, the effect size estimates published were zero (Supplementary Figure 7), moving the pooled effect size closer to the null. We find no statistically significant linear effect of decades since publishing on effect sizes (*b* = 0.10, *p* = .448), showing that over the years the effect sizes have remained generally comparable. Although we visually note an increase in study sample sizes over the years, it was not statistically significant (Supplementary Figure 10). We find no evidence for trends in citation patterns, except for studies with smaller samples getting more cited (Supplementary Figure 15, Supplementary Table 11).

#### Summary

We find an overall positive effect of age on social preference but this effect is smaller than the previous published estimates (*r* = 0.11 vs 0.24; Sparrow et al., 2021). Concerning moderators, we find some evidence for an effect of effect size metric and study design. We find little evidence of temporal trends. Concerning publication bias, additional analyses using Egger’s regression provide some evidence of publication bias (see Supplementary Appendix).

### Effort

#### Meta-analysis

The meta-analysis of 23 effect sizes revealed a positive but not significant effect of age on effort discounting (*r* = 0.24, 95% CI [−0.05, 0.52], *p* = .087). Further, from the equivalence tests, we note that the upper bound equivalence test was nonsignificant (Figure 2A);  therefore, we cannot reject that the association between effort discounting and age is different from 0.1.

We observe substantial heterogeneity, *Q*([*df* = 22] = 132.60, *p* < 0.001), despite the small number of studies included (*s* = 7). We explored the potential moderating role of (a) sample age range, (b) gender, (c) effect size metric, (d) effort type, and (e) domain (Supplementary Table 7). We did not consider incentivization, study context, or study design as moderators because all studies were conducted in a laboratory context, and except for one study involved incentivized decisions and had an extreme group design. Out of the included moderators, effort type had a statistically significant effect on the observed outcomes. Cognitive effort discounting was greater for older than younger adults (*b* = 0.47, 95% CI [0.39, 0.55], *p* = .001).

#### Cumulative meta-analysis and historical trends

Similar to age differences in social preference, age differences in effort discounting have been more recently investigated (Figure 1). Initially, the first article (Westbrook et al., 2013) was published reporting significant age differences (*r* = 0.53, *SE* = 0.07), but subsequent studies provided mixed results. We tested for the presence of the Proteus Phenomenon, and obtained a *z* value of −0.89, *p* = .375, suggesting that the results by Westbrook et al. (2013) did not differ significantly above chance from the other results. Given the small sample size of these studies, the error is wide (Supplementary Figure 7) and the pooled effect size has a quite wide error range. Within the brief time that age differences in effort discounting have been investigated, we find no statistically significant linear effect of decades since publishing on effect sizes (*b* = 0.32, *p* = .333), nor an increase in study sample sizes over the years (Supplementary Figure 10).

#### Summary

We find an overall positive but not significant effect of age on effort preferences. Concerning moderators, there is evidence for the role of effort type (i.e., physical vs cognitive) suggesting that there is an effect of age on effort discounting specific to cognitive effort. Yet, given the small number of studies included in our analysis, further evidence for both types of effort is still required to assess the robustness of this result. Concerning temporal trends, we find no discernible trends in effect sizes or sample sizes. Finally, additional analyses show no evidence of publication bias (see Supplementary Appendix).

## Discussion

We aimed to contribute to a better understanding of the match between extant theoretical accounts of age differences in economic preferences and the associated empirical literature by providing a tabular overview of theories that have been used to make predictions about age differences in economic preferences and conducting a quantitative synthesis of the results of behavioral studies. For this purpose, we conducted systematic literature searches and meta-analyses to estimate overall age effects on risk, time, social, and effort preferences. We also investigated the role of possible moderators, including domain (e.g., gain vs loss), measurement characteristics (e.g., description vs experience, incentivization), and study or sample characteristics (e.g., proportion of females). Furthermore, we assessed historical trends in evidence accumulation through the use of cumulative meta-analysis and by exploring historical trends in research practices (e.g., sample sizes). All in all, we hoped our approach could provide an assessment of the adequacy of different theories of age differences in economic preferences to account for the current and past empirical records.

### Main Findings

Overall, our meta-analyses identified nonsignificant effects of age for risk (*r* = −0.02, 95% CI [−0.06, 0.02]) and effort (*r* = 0.24, 95% CI [−0.05, 0.52]) preferences, and a small but significant effect of age for social (*r* = 0.11, 95% CI [0.01, 0.21]) and time (*r* = −0.04, 95% CI [−0.07, −0.01]) preferences, suggesting increased altruism and patience with age, respectively. More generally, we find all effects are small and cannot be fully distinguished from an equivalence bound of *r* = |0.1|, which can be considered a practically or theoretically meaningful interval.

Taken together, these results suggest either nonexistent or small effects of age in economic preferences. These results are compatible with past meta-analytic work on risk (Best & Charness, 2015; Mata et al., 2011), which did not show an overall effect of age on risk taking in behavioral tasks. For time, our results are similar to those of a previous meta-analysis (Seaman et al., 2022) that reported a small negative, albeit nonsignificant effect of age on temporal discounting. In turn, the results for social preferences are smaller in magnitude than the previous meta-analytic estimate (Sparrow et al., 2021). Finally, the meta-analytic result for effort preferences reflects the mixed findings observed in primary studies of age differences in this area (e.g., Hess et al., 2021; Westbrook et al., 2013).

Concerning the analysis of moderators, our results are particularly noteworthy in the context of risk preferences for which different theories have been proposed that make specific predictions about different moderators. In line with past syntheses (Best & Charness, 2015) and theories that foresee differential age effects as a function of gain and loss domains (cf. Depping & Freund, 2011), we find evidence of age differences in risk preference in the gain relative to the loss domain. Furthermore, contrary to predictions from the confound hypothesis (Frey et al., 2021; Olschewski et al., 2018) and past empirical results (Mata et al., 2011), we do not find a significant pattern of larger age effects in decisions from experience. The main reason for these differences appears to be the inclusion of novel evidence relative to the previous meta-analysis (Mata et al., 2011). Overall, the role of other moderators, such as the use of incentivization, does not seem to account for systematic variance in effect sizes in economic preferences, but some methodological choices (i.e., correlation type and study design) do account for some variance in the social and time preference domain. Furthermore, for temporal discounting, we observe an age difference in online relative to laboratory studies: Laboratory and online studies may differ in their sample characteristics and it would be interesting to assess the extent to which sample composition (e.g., education level) accounts for such differences in future work.

Concerning historical trends, the apparent visual trend across economic preferences is for effect sizes to approach zero over time; however, we found overall no evidence of significant effects over time for either effect sizes or research practices as quantified by sample size of the studies conducted. As noted in earlier work (Seaman et al., 2022), the results for time preference make clear that the overall null effect of age on temporal discounting was already apparent early in the research history of the topic, because the large effect reported in the seminal paper was not replicated in subsequent studies (Green et al., 1994). More broadly, one should note that the four types of economic preferences differ considerably in the number of effect sizes available for analysis (193, 125, 28, and 23, for risk, time, social, and effort preferences, respectively), suggesting it could be important to assess the development of such trends in future work, particularly for the social and effort preferences for which comparatively little evidence is available.

Finally, concerning our analyses of publication bias, *p*-curve analyses found no evidence of *p*-hacking but we found evidence of a Proteus effect (i.e., the tendency for early replications of a scientific work to contradict the original findings; Ioannidis & Trikalinos, 2005; Koricheva et al., 2013; Young et al., 2008) in the time preferences literature and Egger’s regression provided some ground to suspect systematic publication bias in the social preferences literature. These results do not fully assuage concerns surrounding the overestimation of age effects in the aging literature (Isaacowitz, 2020), but also do not provide evidence for widespread publication bias.

### Implications

All in all, our results have some major theoretical and methodological implications. First and foremost, concerning theory, our finding of small to null age effects detected across the empirical literature questions the adequacy of many extant theories that predict age differences in economic preferences. One direct consequence is that the theoretical perspectives concerning risk preferences need to be revised. Indeed, our results reject theories that posit a strong role for cognitive and learning effects (cf. Mata et al., 2011), but provide support for theories predicting differential age effects as a function of gain and loss domains (cf. Depping & Freund, 2011). We propose that future theorizing should focus more specifically on the mechanisms thought to underlie age differences (e.g., dopaminergic function, time horizon) and empirical work should aim to provide critical tests of the role of such mechanisms (cf. Frey et al., 2015; Zilker & Pachur, 2021) rather than simply assess a directional effect of age. It may also be important to distinguish critical claims of theories, such as the age trends associated with specific mechanisms, and auxiliary assumptions, such as the role of task or measurement characteristics (e.g., role of incentivization, task complexity). We discuss the specific point concerning assumptions about operationalization in the Limitations section below.

Second, concerning methodological implications, the few indications of publication bias suggest future work may want to consider different sources of bias and the use of registered reports to correct our estimates of age differences in economic preferences.

Third, and more broadly, even though we could not distinguish clear-cut phases in the development of the research topic, we would like to encourage researchers studying aging to integrate cumulative approaches in their work. Here, we focused on economic preferences but this approach could be extended to other central constructs in aging research, such as memory performance, executive functioning, or well-being. In doing so, we could detect areas in which age differences are more established, robust, and stable than others, which, ultimately, could improve how we justify the need for additional research, how resources are allocated, and how participants are recruited.

To summarize, our meta-analysis did not find evidence to support the predictions made by the theories that are most frequently discussed in the literature on aging and preferences for risk and effort. For time preference, more than half of these theories (e.g., dopaminergic neuromodulation hypothesis) predict a decrease in temporal discounting with age; however, given that effect we identified is of very small magnitude, the extent to which these theories are supported is questionable. When it comes to social preference, our results suggest that there is a small increase in altruism with age, which is consistent with the predictions made by close to all the theories that we examined in this domain. However, there are relatively few studies concerning social and effort preferences, and our results do not provide sufficient evidence to distinguish between the various mechanisms proposed suggesting more work is needed in the area of economic preferences.

### Limitations and Future Directions

We should also point out some limitations of our work. First, a wide range of measures has been developed to quantify individuals’ economic preferences (Charness et al., 2013; Eckel, 2019). In the present study, we focus solely on behavioral tasks, yet self-reported measures (e.g., propensity measures) could also be considered. The convergent validity of different measures within each preference is low (Duckworth & Kern, 2011; Frey et al., 2017; Levitt & List, 2007; Strand et al., 2018), which suggests further research should focus on the comparability of effect size trajectories across different measurement types. For example, recent work suggests that self-reports are more likely to capture systematic age differences in risk preference (Frey et al., 2021) and a recent quantitative synthesis suggests robust age effects when considering self-report measures (Liu et al., 2023). Although past theorizing has largely ignored the role of measurement, the differences between our results and those for self-reported risk propensity (Liu et al., 2023) suggest that it would be important to develop more specific expectations about the role of operationalization in detecting age differences in economic preferences.

Second, our work focused solely on published results because of our aim of assessing the historical patterns in the literature. However, published results are unlikely to be fully representative of the evidence on age differences thus data from unpublished reports or data sets could be included in future extensions of this work.

Third, although we considered a wide range of moderators to explain effect-size heterogeneity, cultural and sociodemographic factors (e.g., education) were not included. Details on such factors are often missing in primary studies or reported heterogeneously, which can be challenging to incorporate in analyses. However, as such factors can influence economic preferences (cf. Frey et al., 2021), this can be an avenue for future research.

Lastly, we did not preregister this work. We note, however, that we make all the data and code used in this study publicly available to ensure that our work can be assessed transparently and used in future confirmatory efforts.

## Conclusion

Our results indicate that age differences in economic preferences as captured by behavioral tasks are not as pervasive as extant theories would imply and that more specific theorizing is needed to make predictions for different preference types (risk, time, social, effort) and their operationalizations.

## Supplementary Material

gbad034_suppl_Supplementary_Material

## Data Availability

This study was not preregistered, but all the data and scripts are publicly available in an online repository (https://github.com/cdsbasel/cumulative).

## Footnotes

Sparrow et al. (2021) reported an overall effect of *g* = 0.61 (*r* = 0.31; 95% CI [0.25, 0.37]; *p* < .001); however, one of the outcomes used in their analyses was coded in the opposite, incorrect direction (https://osf.io/9hacs). Upon correction, the mean effect size becomes *g* = 0.48 which we converted into a correlation coefficient (*r* = 0.24; 95% CI [0.12, 0.35]; *p* = .001).
