# Supplementary material for: Aging and Economic Preferences: Cumulative Meta-Analyses of Age Differences in Risk, Time, Social, and Effort Preferences
Source: J Gerontol B Psychol Sci Soc Sci. 2023 Mar 4;78(7):1122–35. doi: 10.1093/geronb/gbad034 (PMC10292840; doi:10.1093/geronb/gbad034)
Supplement: gbad034_suppl_Supplementary_Material [file gbad034_suppl_supplementary_material.pdf]

Supplementary Appendix

Aging and Economic Preferences: Cumulative meta-analyses of age differences in risk, time, social, and effort preferences

Alexandra Bagaïni, M.Sc.[1], Yunrui Liu, M.Sc.[1], Arzie Bajrami, B.Sc.[1], Gayoung Son, B.Sc.[1], Loreen Tisdall, Ph.D.[1], and Rui Mata, Ph.D.[1]

[1]Center for Cognitive and Decision Sciences, University of Basel

Version date: February 23, 2023

Supplementary Appendix

Aging and Economic Preferences: Cumulative meta-analyses of age differences in risk, time, social, and effort preferences

Supplementary Methods

Scoping Review: Literature Search

We first conducted a computerized literature search of publication records on Web of Science to identify previous meta-analyses of age differences in either risk, time, social, or effort preferences. We searched for publications published until November 1st, 2022 that pertained to the specified search terms (Table S2). From our search for meta-analytical studies, we selected those that reported findings on (a) behavioral tasks involving monetary transactions (real or hypothetical), (b) the adult population (i.e., 18 years or above) and (c) economic preferences that met the definitions from Table 1. We followed the Preferred Reporting Items for Systematic Reviews and Meta-Analyses (PRISMA) guidelines (Page et al., 2021), and details of this search and screening process are available in Figure S1. In a second step, we examined more closely the search strategy, the eligibility criteria and the included studies of the selected meta-analytical studies to inform our search and screening of primary studies. We noted that the meta-analyses identified via the scoping review were heterogeneous, notably with regards to year of publication and eligibility criteria. Therefore, we adapted our search strategy of individual studies such that the meta-analyses could be comparable across economic preferences.

Publication Bias

To explore evidence of publication bias (i.e., tendency to publish only significant effects), we produced for each set of effect sizes a contour-enhanced funnel plot (Peters et al., 2008). This plot displays the distribution of effect sizes against a precision metric (Figure S13). To assess the absence or presence of publication bias in each set of effect sizes, we visually inspected the funnel plots and conducted multilevel Egger's regression tests (Rodgers & Pustejovsky, 2021). For the Egger's tests, we fitted three-level

meta-regression models with different precision metrics, one with standard error and another with the inverse sample size as predictor. In addition, we conducted p-curve tests (Simonsohn et al., 2014) using the dmetar package (Harrer et al., 2019). This test is used to detect evidence of p-hacking; that is, researchers selectively choosing or analyzing data such that non-significant effects become significant (Figure S14). Only effect sizes significant at alpha = 5% level, which determine whether the distribution of p-values is right-skewed distribution and whether studies are properly powered, are included in these analyses (Simonsohn et al., 2014).

Citations

We investigated how the impact of publications on age differences in economic preferences changed over time, and the association between citations and effect sizes and sample sizes. First, for each publication we obtained the number of citations it received every year since it was published, including its citations as a pre-print. Then, for each preference we fitted two linear models, to assess (a) the effect of a publication's average sample size (log-transformed), and (b) aggregated effect size (accounting for effect size dependency) on the median yearly citations (log-transformed), while controlling for the number of decades it has been available (either as a published article or as a pre-print). For these analyses, we included a total of 120 publications (risk = 54, time = 48, social = 12, effort = 6). We excluded publications (n = 6: risk = 3, time = 2, social = 1) for which yearly citation information was not available from Google Scholar.

Supplementary Results

Publication Bias

Risk. From the visual inspection of the funnel plot (Figure S13) and the results of the multilevel Egger's test, there is no evidence of publication bias using either standard error or the inverse sample size as the precision metric (Table S8) nor was there any evidence of p-hacking from the p-curve test. The right skew analyses were all significant, and the flatness tests were all non-significant (Table S9). Lastly, the power

to detect an effect exceeded 80% textcolorred(power = 94%, 95% CI [90.7%, 96.7%]).

Time. results of the multilevel Egger's test showed no evidence of publication bias. (Table S8). P-curve test results suggested no evidence of p-hacking (Table S9), and studies were overall sufficiently powered to detect an effect textcolorred(power = 84% [73.4%-90.8%]).

Social. The multilevel Egger's test results vary depending on the precision metric used: Using standard error as a precision metric we note no significant asymmetry, whereas an assymetry is detected when using the inverse sample size as a predictor (Table S8). When inspecting the p-curve results, we find no evidence of p-hacking (Table S9) and the power to detect an effect on average exceeds 80% textcolorred(power = 83%, 95% CI [58.9%, 94.5%]).

Effort. The results of the multilevel Egger's test showed no evidence of publication bias. (Table S8). P-curve test results suggested no evidence of p-hacking (Table S9), and studies were overall sufficiently powered to detect an effect (power = 99% [97.2%-99%]).

Citations

Figure S15A shows, for each preference, the number of yearly citations of each publication as a function of the number of years it has been published. Figures S15B and S15C show, for each preference, the relation between median yearly citations and the publication's aggregated effect size and average sample size, respectively. Tables S10 and S11 summarize the results from the linear regressions on the association between median yearly citations with (a) effect sizes and (b) sample sizes, respectively.

Risk. We find no significant effect of effect size on the median number of yearly citations. However, older publications and publications with larger samples are more often cited.

Time. There is no significant effect of effect size or sample size on the median number of yearly citations. However, older publications are more often cited.

Social. We note no significant effect of effect size on the median number of yearly citations, but older publications and publications with smaller samples are more

often cited.

Effort. We find no significant effect of effect size or sample size on the median number of yearly citations.

All in all, the results show no evidence that larger effect sizes have received more attention in the literature in the form of citations, which reduces concerns that studies finding larger age differences had a stronger impact in shaping the aging literature on economic preferences.

References

Depping, M. K., & Freund, A. M. (2011). Normal aging and decision making: The role of motivation. *Human Development*, *54*(6), 349–367. https://doi.org/10.1159/000334396

Frey, R., Richter, D., Schupp, J., Hertwig, R., & Mata, R. (2021). Identifying robust correlates of risk preference: A systematic approach using specification curve analysis. *Journal of Personality and Social Psychology*, *120*(2), 538–557. https://doi.org/10.1037/pspp0000287

Harrer, M., Cuijpers, P., Furukawa, T., & Ebert, D. D. (2019). Dmetar: Companion R package for the guide "Doing meta-analysis in R".

Harrison, G. W. (1994). Expected Utility Theory and the Experimentalists. In J. D. Hey (Ed.), *Experimental Economics* (pp. 43–73). Physica-Verlag HD. https://doi.org/10.1007/978-3-642-51179-0_4

Jacobs, P., & Viechtbauer, W. (2017). Estimation of the biserial correlation and its sampling variance for use in meta-analysis. *Research Synthesis Methods*, *8*(2), 161–180. https://doi.org/10.1002/jrsm.1218 _eprint: https://onlinelibrary.wiley.com/doi/pdf/10.1002/jrsm.1218

Olschewski, S., Rieskamp, J., & Scheibehenne, B. (2018). Taxing cognitive capacities reduces choice consistency rather than preference: A model-based test. *Journal of experimental psychology. General*, *147*, 462–484. https://doi.org/10.1037/xge0000403

Page, M. J., McKenzie, J. E., Bossuyt, P. M., Boutron, I., Hoffmann, T. C., Mulrow, C. D., Shamseer, L., Tetzlaff, J. M., Akl, E. A., Brennan, S. E., Chou, R., Glanville, J., Grimshaw, J. M., Hróbjartsson, A., Lalu, M. M., Li, T., Loder, E. W., Mayo-Wilson, E., McDonald, S., … Moher, D. (2021). The PRISMA 2020 statement: An updated guideline for reporting systematic reviews. *British Medical Journal*, *372*, n71. https://doi.org/10.1136/bmj.n71

Peters, J. L., Sutton, A. J., Jones, D. R., Abrams, K. R., & Rushton, L. (2008). Contour-enhanced meta-analysis funnel plots help distinguish publication bias

from other causes of asymmetry. *Journal of Clinical Epidemiology*, *61*(10), 991–996. https://doi.org/10.1016/j.jclinepi.2007.11.010

Pustejovsky, J. E. (2014). Converting from d to r to z when the design uses extreme groups, dichotomization, or experimental control. *Psychological Methods*, *19*(1), 92–112. https://doi.org/10.1037/a0033788

Rodgers, M. A., & Pustejovsky, J. E. (2021). Evaluating meta-analytic methods to detect selective reporting in the presence of dependent effect sizes. *Psychological Methods*, *26*(2), 141–160. https://doi.org/10.1037/met0000300

Sassenberg, K., & Ditrich, L. (2019). Research in social psychology changed between 2011 and 2016: Larger sample sizes, more self-report measures, and more online studiesures, and More Online Studies. *Advances in Methods and Practices in Psychological Science*, *2*(2), 107–114. https://doi.org/10.1177/2515245919838781

Simonsohn, U., Nelson, L. D., & Simmons, J. P. (2014). P-curve: A key to the file-drawer. *Journal of Experimental Psychology: General*, *143*(2), 534–547. https://doi.org/10.1037/a0033242

Smith, V. L., & Walker, J. M. (1993). Monetary rewards and decision cost in experimental economics. *Economic Inquiry*, *31*(2), 245. https://doi.org/10.1111/j.1465-7295.1993.tb00881.x

Table S1

Overview of moderators included in the meta-regressions.

Moderator	Description	Rationale
Study Design	Two levels: Extreme group design (e.g., young and older adult groups) vs. continuous (e.g., participants aged 18 to 70)	Accounting for potentially more extreme results in studies using extreme group designs
Effect size metric	Two levels: Pearson's r vs. point-biserial correlation	These metrics have different underlying assumptions (Jacobs & Viechtbauer, 2017; Pustejovsky, 2014)
Incentivization	Two levels: hypothetical vs. incentivized	May influence participant's motivation, and may affect the extent that participants reveal their preferences (Harrison, 1994; Smith & Walker, 1993)
Task format	Two levels: description vs. experience (only applicable to risk preferences)	Confound hypothesis (e.g., Frey et al., 2021; Olschewski et al., 2018)
Domain	Three levels: gain vs. loss vs. mixed (only applicable to risk and effort preference)	Motivational theories (e.g., Depping & Freund, 2011)
Study context	Two levels: online vs. in-person	Accounting for changes in how studies are being conducted and how it allows to collect data from larger and more representative samples (e.g., Sassenberg & Ditrich, 2019)
Gender	Proportion of females in the sample	Past literature suggests gender differences in at least some operationalizations of economic preferences (cf. Frey et al., 2021)
Age range	Differences in decades between the young and old sample	A wider age range may increase power to detect age differences

Table S2

Search terms used to conduct the computerized literature searches on Web of Science.

Section	Search Terms
Meta-Analyses of Aging and Preferences	*(age OR aging OR ageing OR "older adults") AND ("risky choice" OR "risk taking" OR "risk-taking" OR altruis* OR prosoci* OR philanthrop* OR generativity OR framing OR "prospect theory" OR "dictator game" OR "delay aversion" OR "delay of gratification" OR "social preference" OR "risk aversion" OR "time preference" OR "intertemporal choice" OR "temporal discounting" OR "delay discounting" OR "effort discounting" OR "effort-based decision" OR "effort-based choice") AND ("meta analysis" OR "meta-analysis")*
Risk	*(age OR aging OR ageing OR "older adults") AND (risky OR "risky choice" OR "risk taking" OR "risk-taking" OR framing OR "prospect theory") AND ("decision making")*
Time	*(age OR aging OR ageing OR "older adults") AND ("temporal discounting" OR "intertemporal" OR "delay discounting" OR "inter-temporal" OR "delay aversion" OR "delay of gratification" OR "delay gratification" OR "time preference*") AND ("choice*" OR "task" OR "decision" OR "game" OR "procedure" OR "measure" OR "paradigm")*
Social	*(age OR aging OR ageing OR "older adults") AND ("altruis*" OR "social*" OR "prosocial*") AND ("dictator game" OR "discounting" OR "moral decision" OR "giving game" OR "economic decision")*
Effort	*(age OR aging OR ageing OR "older adults") AND ("effort") AND (discount* OR decision OR choice OR "tradeoff" OR "trade off" OR "cost-benefit") AND (task OR exert OR game OR paradigm)*

Table S3

Primary study eligibility criteria.

**Aspect**	**Preference**			
	Risk	Social	Time	Effort
Definition	Decision under risk only, not confounded by a social, time or effort dimension.	Altruism/prosocial decisions only, not confounded by a time, risk or effort dimension.	Decision involving a delay, not confounded by a social, risk or effort dimension.	Decision involving effort, not confounded by a social, temporal or risk dimension.
Domain	Gain, loss and mixed domain	Not applicable	Gain domain only	Gain, loss and mixed domain
Type of measure	Studies with a behavioral measure involving money/rewards (real or hypothetical)			
Decision environment	Studies completed in a laboratory or online or controlled setting. We exclude behavior collected in an MRI scanner, during EEG measurements or in the context of a pharmacological study.			
Population	Healthy adults			
Age	Adults (i.e., majority of participants are at least 18 years old). Sample needs to have an age range of at least 25 years, (i.e., difference between the maximum and minimum age)			
Type of study	Empirical study. Longitudinal or cross-sectional study			
Type of DV	Numerical or graphical format of results. Quantitative value of age differences with data either for each age group or on the relation between behavior and age (e.g., correlation). Excludes categorical outcomes. Data collected under conditions that should be free of experimental manipulations that would result in a confound (e.g., participants shown a prime prior to making decisions)			

Table S4

Three-level meta-regression results (rho = .5)[1] *with effect sizes of primary studies on age differences in risk preference (k = 193).*

Reg.#	Moderator	Estimate	SE	t-val	p-val	95% CI
1	Age range	<0.001	0.014	0.035	0.972	[-0.029, 0.03]
2	Prop. female	-0.03	0.235	-0.126	0.902	[-0.562, 0.503]
3	Metric-Correlation	-0.016	0.019	-0.864	0.396	[-0.055, 0.023]
3	Metric-Point-Biserial corr.	-0.03	0.033	-0.891	0.379	[-0.097, 0.038]
4	Study-Age continuous	-0.038	0.02	-1.901	0.076	[-0.081, 0.005]
4	Study-Extreme group	-0.018	0.027	-0.67	0.507	[-0.074, 0.037]
5	Incentivization-Hypothetical	-0.024	0.025	-0.983	0.339	[-0.076, 0.028]
5	Incentivization-Incentivized	0.002	0.028	0.088	0.93	[-0.054, 0.058]
6	Domain-Gain	-0.054	0.023	-2.354	0.023	[-0.101, -0.008]
6	Domain-Loss	0.019	0.044	0.42	0.679	[-0.074, 0.111]
6	Domain-Mixed	0.012	0.036	0.345	0.734	[-0.063, 0.088]
7	Task-Description	-0.015	0.024	-0.634	0.529	[-0.064, 0.034]
7	Task-Experience	-0.051	0.039	-1.288	0.217	[-0.134, 0.033]
8	Context-In-person	-0.03	0.026	-1.157	0.254	[-0.082, 0.022]
8	Context-Online	-0.004	0.023	-0.178	0.862	[-0.054, 0.046]

Reporting cluster-robust standard errors and confidence intervals.

[1] Results were not substantially affected by varying rho values (i.e., correlation between sampling errors within studies).

Table S5

Three-level meta-regression results (rho = .5)[1] with effect sizes of primary studies on age differences in time preference (k = 125).

Reg.#	Moderator	Estimate	SE	t-val	p-val	95% CI
1	Age range	0.015	0.01	1.452	0.16	[-0.006, 0.035]
2	Prop. female	0.035	0.150	0.230	0.822	[-0.287, 0.356]
3	Metric-Correlation	-0.042	0.016	-2.694	0.009	[-0.074, -0.011]
4	Metric-Point-Biserial corr	-0.025	0.056	-0.446	0.662	[-0.145, 0.095]
5	Study-Age continuous	-0.045	0.016	-2.878	0.006	[-0.077, -0.014]
5	Study-Extreme group_group	-0.015	0.054	-0.274	0.788	[-0.129, 0.099]
6	Incentivization-Hypothetical	-0.034	0.016	-2.148	0.035	[-0.065, -0.002]
6	Incentivization-Incentivized	-0.087	0.089	-0.973	0.362	[-0.297, 0.123]
7	Context-In-person	-0.014	0.038	-0.386	0.702	[-0.091, 0.062]
7	Context-Online	-0.052	0.016	-3.307	0.002	[-0.083, -0.02]

Reporting cluster-robust standard errors and confidence intervals.

[1] Results were not substantially affected by varying rho values (i.e., correlation between sampling errors within studies).

Table S6

Three-level meta-regression results (rho = .5)[1] *with effect sizes of primary studies on age differences in social preference (k = 28).*

Reg.#	Moderator	Estimate	SE	t-val	p-val	95% CI
1	Age range	-0.059	0.046	-1.274	0.241	[-0.166, 0.049]
2	Prop. female	0.031	0.257	0.12	0.913	[-0.867, 0.929]
3	Metric-Correlation	0.054	0.075	0.724	0.491	[-0.121, 0.23]
3	Metric-Point-Biserial corr.	0.17	0.05	3.364	0.011	[0.052, 0.287]
4	Study-Age continuous	0.123	0.05	2.476	0.043	[0.005, 0.242]
4	Study-Extreme group	0.103	0.078	1.319	0.221	[-0.075, 0.28]
5	Incentivization-Hypothetical	0.113	0.082	1.372	0.205	[-0.074, 0.3]
5	Incentivization-Incetivized	0.109	0.048	2.268	0.059	[-0.006, 0.223]
5	Context-In-person	0.121	0.056	2.134	0.053	[-0.002, 0.243]
5	Context-Online	0.085	0.097	0.877	0.447	[-0.227, 0.396]

Reporting cluster-robust standard errors and confidence intervals.

[1] Results were not substantially affected by varying rho values (i.e., correlation between sampling errors within studies).

Table S7

Three-level meta-regression results (rho = .5)[1] with effect sizes of primary studies on age differences in effort discounting (k = 23).

Reg.#	Moderator	Estimate	SE	t-val	p-val	95% CI
1	Age range	-0.121	0.217	-0.56	0.616	[-0.825, 0.582]
2	Prop. female	-1.957	2.709	-0.723	0.542	[-13.114, 9.2]
3	Metric-Correlation	0.054	0.193	0.281	0.805	[-0.776, 0.884]
3	Metric-Point-Biserial corr.	0.36	0.128	2.8	0.069	[-0.052, 0.771]
4	Effort Type-Cognitive	0.469	0.023	20.35	0.001	[0.385, 0.552]
4	Effort Type-Physical	-0.086	0.056	-1.533	0.272	[-0.342, 0.17]
5	Domain-Gain	0.252	0.112	2.258	0.065	[-0.021, 0.526]
5	Domain-Loss	0.061	0.106	0.581	0.584	[-0.201, 0.324]

Reporting cluster-robust standard errors and confidence intervals.

[1] Results were not substantially affected by varying rho values (i.e., correlation between sampling errors within studies).

Table S8

Egger's regression test results with effect sizes of primary studies on age differences in risk (k = 193), time (k = 125), social (k = 28) and effort (k = 23) preference. Three-level meta-regression with standard error or inverse sample size as a predictor.

Precision	Estimate	SE	t-val	p-val	95% CI
		Risk			
Standard error	-0.033	0.4	-0.083	0.934	[-0.859, 0.792]
Inverse Sample Size	1.035	3.836	0.27	0.79	[-6.942, 9.012]
		Time			
Standard error	-0.697	0.374	-1.861	0.071	[-1.456, 0.063]
Inverse Sample Size	-4.133	2.536	-1.63	0.115	[-9.338, 1.072]
		Social			
Standard error	3.033	1.987	1.527	0.163	[-1.5, 7.566]
Inverse Sample Size	16.078	6.758	2.379	0.043	[0.656, 31.499]
		Effort			
Standard error	-1.323	2.434	-0.543	0.638	[-11.049, 8.404]
Inverse Sample Size	16.249	13.346	1.218	0.315	[-27.641, 60.14]

Reporting cluster-robust standard errors and confidence intervals.

Table S9

P-curve analysis results. (R)ight-(S)kewness and flatness test for effect sizes of primary studies on age differences in risk (k = 193), time (k = 125), social (k = 28) and effort (k = 23) preference.

Test name	pBinomial	zFull	pFull	zHalf	pHalf	kFull	kHalf
			Risk				
Right-S test	< 0.001	-17.64	< 0.001	-17.465	< 0.001	65(33.7%)	55(28.5%)
Flatness test	0.996	11.417	> 0.999	17.19	> 0.999	65(33.7%)	55(28.5%)
			Time				
Right-S test	0.001	-10.348	< 0.001	-11.354	< 0.001	40(32%)	30(24%)
Flatness test	0.746	5.728	> 0.999	11.222	> 0.999	40(32%)	30(24%)
			Social				
Right-S test	0.212	-5.166	< 0.001	-5.135	< 0.001	14(50%)	9(32.1%)
Flatness test	0.37	2.899	0.998	6.721	> 0.999	14(50%)	9(32.1%)
			Effort				
Right-S test	< 0.001	-11.293	< 0.001	-10.422	< 0.001	11(47.8%)	11(47.8%)
Flatness test	> 0.999	8.061	> 0.999	9.152	> 0.999	11(47.8%)	11(47.8%)

Table S10

Linear regression analysis results for the association between median yearly citations (log scale) and aggregated effect sizes, controlling for the number of years a publication has been cited. Separate results for risk (publications = 54), time (publications = 48), social (publications = 12) and effort (publications = 6) preference.

Predictor	Estimate	SE	t-val	p-val	95% CI
			Risk		
Intercept	0.5	0.046	10.759	<0.001	[0.409, 0.592]
Effect size	-0.074	0.153	-0.484	0.629	[-0.376, 0.227]
Decades in-print	0.142	0.025	5.632	<0.001	[0.093, 0.192]
			Time		
Intercept	0.442	0.037	11.929	<0.001	[0.369, 0.515]
Effect size	0.067	0.086	0.775	0.439	[-0.102, 0.235]
Decades in-print	0.293	0.021	14.232	<0.001	[0.253, 0.334]
			Social		
Intercept	0.261	0.098	2.662	0.01	[0.065, 0.457]
Effect size	0.252	0.248	1.016	0.314	[-0.245, 0.749]
Decades in-print	0.564	0.102	5.506	<0.001	[0.359, 0.769]
			Effort		
Intercept	0.518	0.121	4.283	<0.001	[0.268, 0.768]
Effect size	0.303	0.241	1.259	0.221	[-0.195, 0.801]
Decades in-print	0.636	0.136	4.69	<0.001	[0.356, 0.917]

Table S11

Linear regression analysis results for the association between a publication's median yearly citation count (log scale) and its average sample size, controlling for the number of years the publication has been cited. Separate results for risk (publications = 54), time (publications = 48), social (publications = 12) and effort (publications = 6) preference.

Predictor	Estimate	SE	t-val	p-val	95% CI
			Risk		
Intercept	0.026	0.123	0.215	0.83	[-0.215, 0.268]
Sample size (log)	0.195	0.047	4.159	<0.001	[0.103, 0.287]
Decades in-print	0.183	0.026	7.009	<0.001	[0.132, 0.234]
			Time		
Intercept	0.368	0.079	4.645	<0.001	[0.212, 0.524]
Sample size (log)	0.031	0.027	1.132	0.258	[-0.023, 0.084]
Decades in-print	0.293	0.02	14.837	<0.001	[0.255, 0.332]
			Social		
Intercept	0.845	0.284	2.978	0.004	[0.277, 1.413]
Sample size (log)	-0.253	0.121	-2.095	0.041	[-0.494, -0.011]
Decades in-print	0.508	0.102	4.996	<0.001	[0.304, 0.711]
			Effort		
Intercept	0.694	0.761	0.912	0.371	[-0.88, 2.269]
Sample size (log)	-0.085	0.38	-0.223	0.826	[-0.87, 0.701]
Decades in-print	0.693	0.148	4.679	<0.001	[0.387, 1]

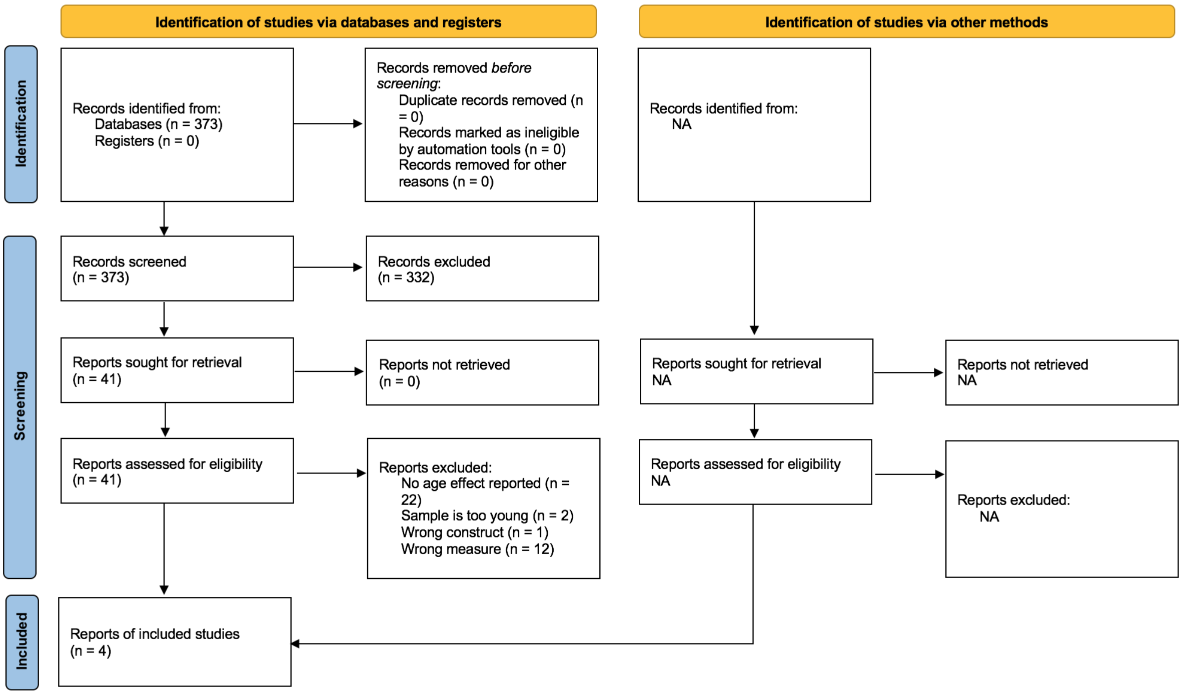

Figure S1

PRISMA flow diagram of the selection process of research synthesis on economic preferences and aging.

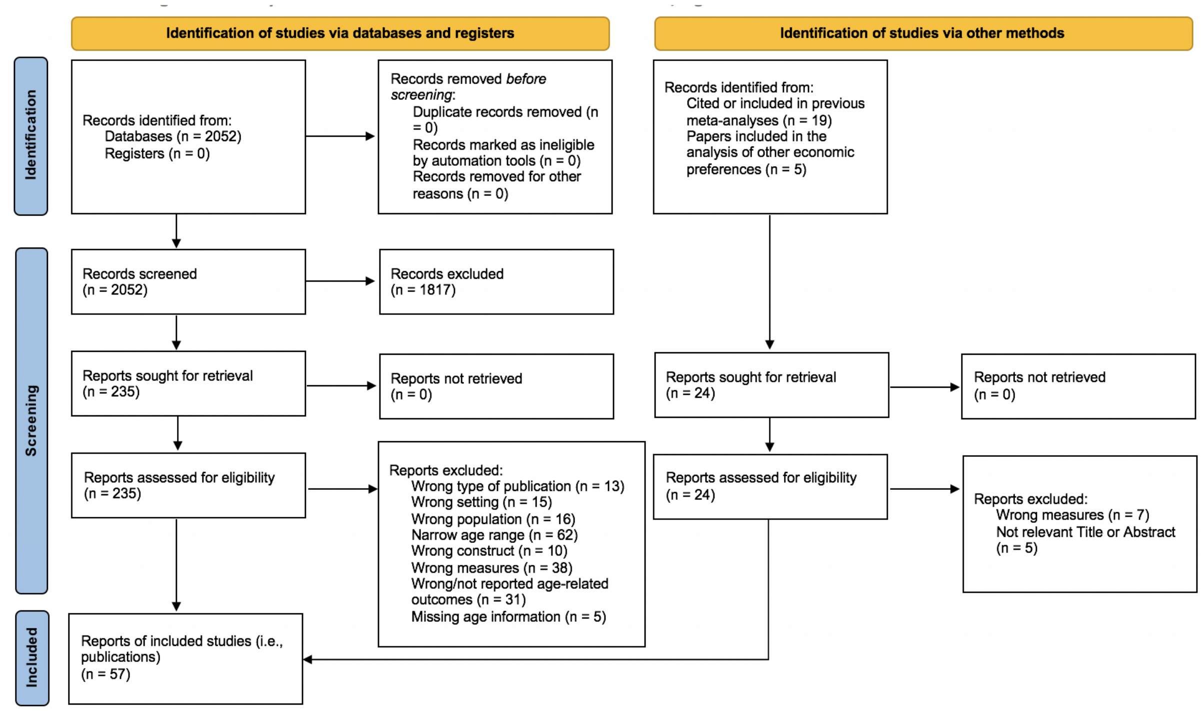

Figure S2

PRISMA flow diagram of the selection process of individual studies on the association between risk preference and age.

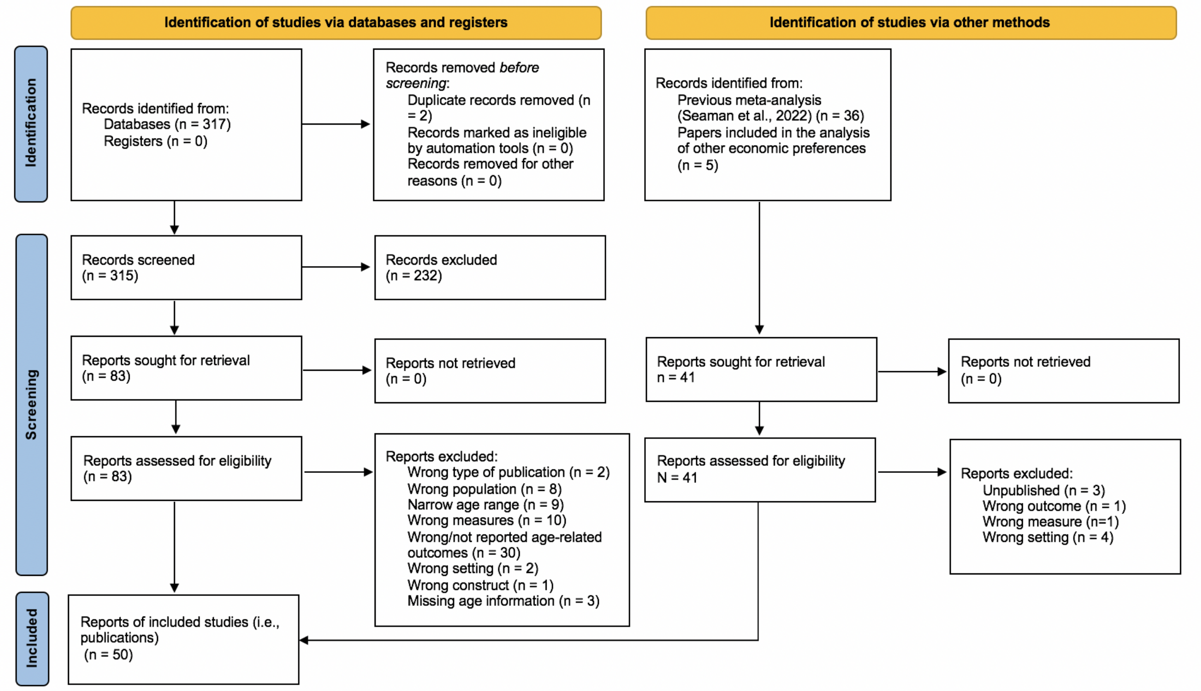

Figure S3

PRISMA flow diagram of the selection process of individual studies on the association between time preference and age.

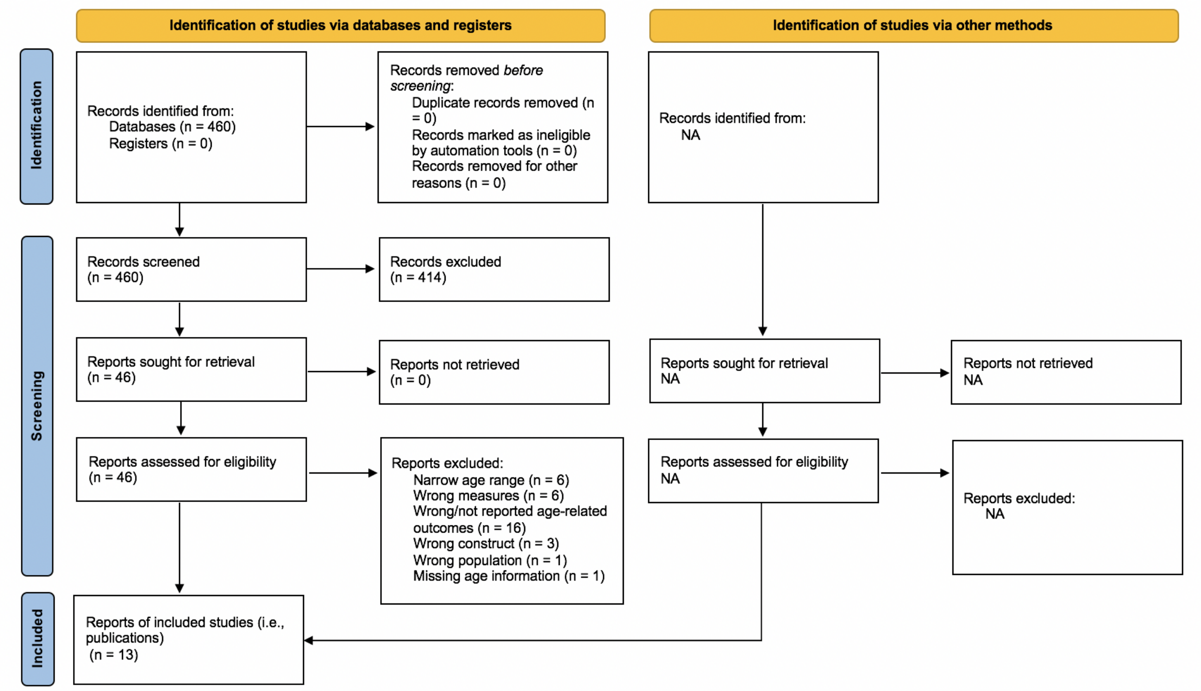

Figure S4

PRISMA flow diagram of the selection process of individual studies on the association between social preference and age.

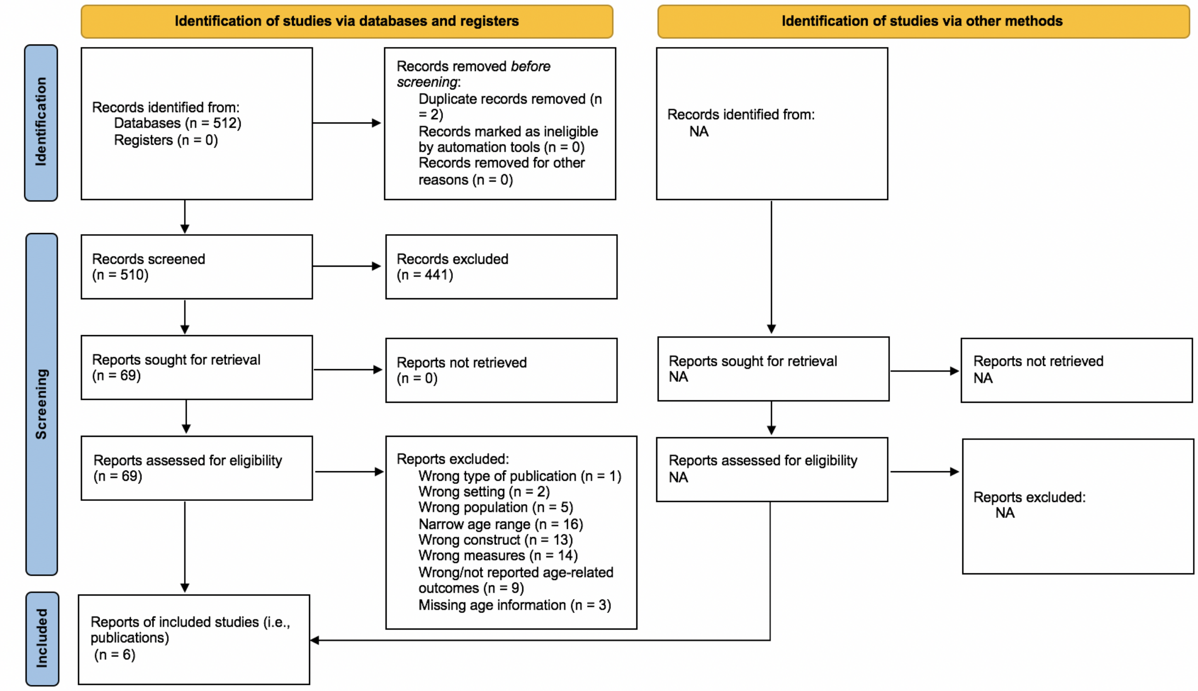

Figure S5

PRISMA flow diagram of the selection process of individual studies on the association between effort-related preference and age.

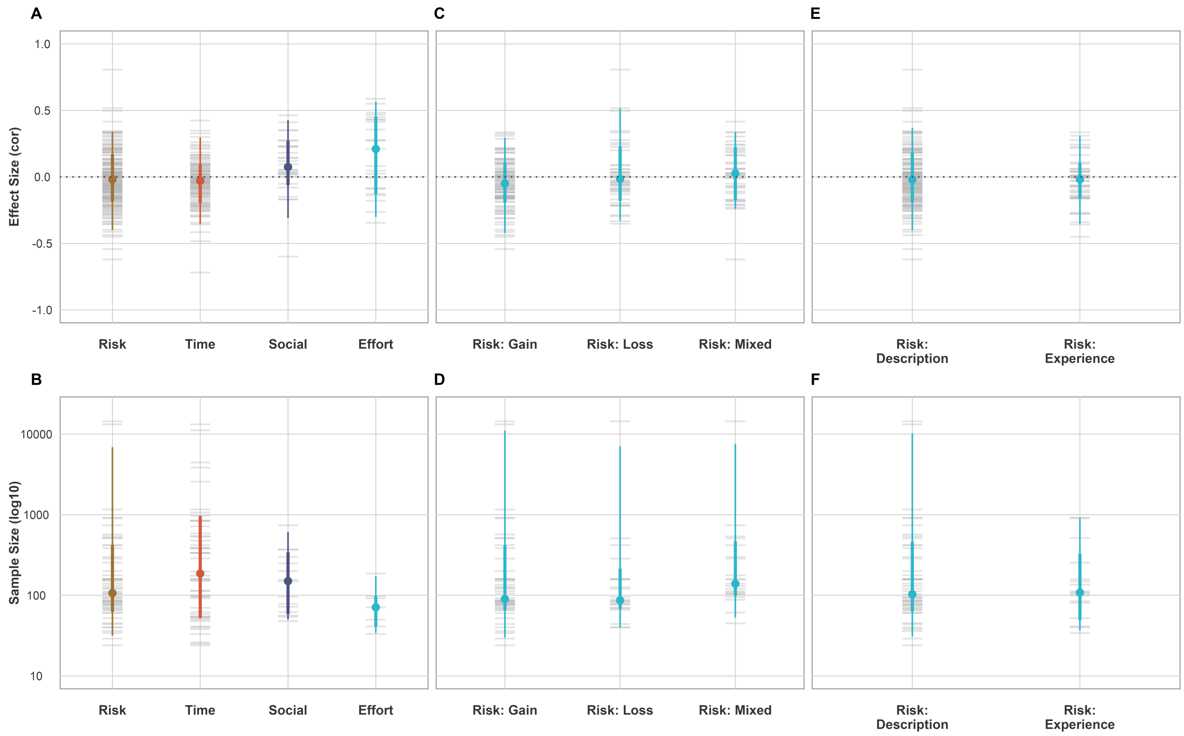

Figure S6

Overview of effect sizes and sample sizes. Grey dashes represent an individual effect size or study, with the overall median, and the 66% and 95% CI. A) Distribution of individual age effects by preference (risk (k = 193), time (k = 125), social (k = 28), and effort (k = 23)). B) Distribution of study sample sizes by preference (risk (studies = 62), time (studies = 54), social (studies = 15), and effort (studies = 7)). C) Distribution of individual age effects by risk preference domain (gain (k = 106), loss (k = 46) and mixed (k = 41)). D) Distribution of study sample sizes by risk preference domain (gain (studies = 48), loss (studies = 22) and mixed (k = 21)). E) Distribution of age effects by risk-taking task category (description (k = 147) and experience (k = 44)). F) Distribution of study sample sizes by risk-taking task category (description (studies = 51) and experience (studies = 17))

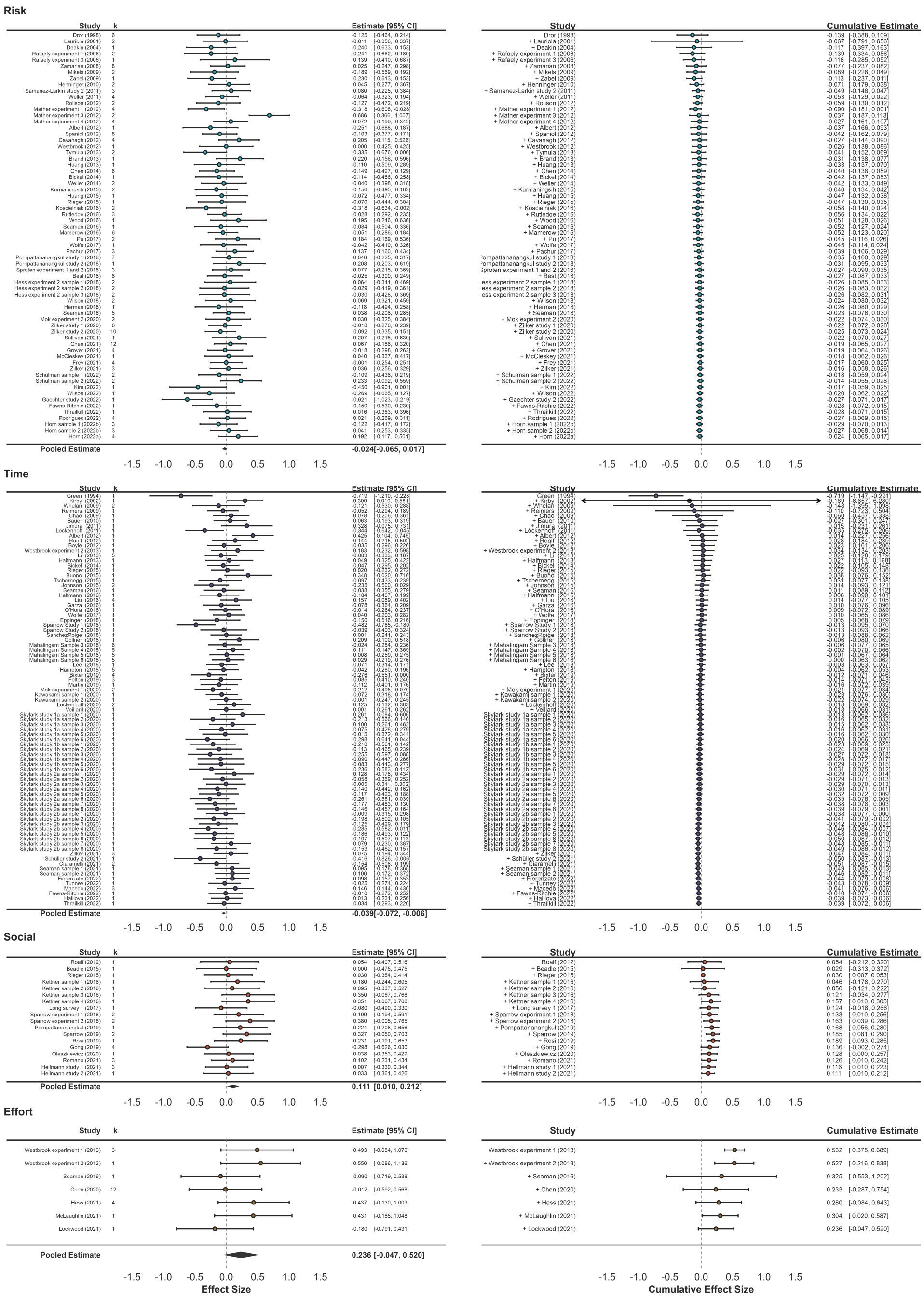

Figure S7

Forest plots of effect sizes on the association between age and risk (k = 193), time (k = 125), social (k = 28), and effort (k = 23) preference. Aggregated forest plots of the three-level meta-analytic model with effect sizes pooled by study with 95% CI, ordered by publication year. Cumulative effect sizes and 95% CI by adding studies in order of publication year.

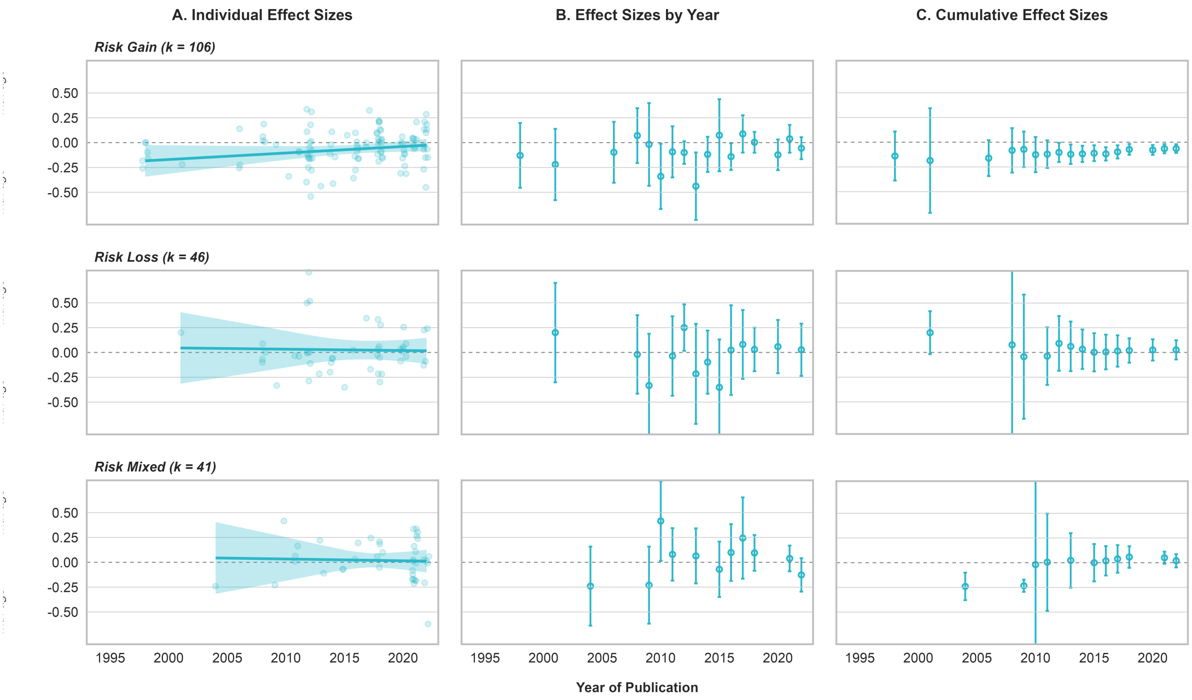

Figure S8

Meta-analytic results of the effect of age on risk taking in the gain (k = 106), loss (k = 46) and mixed (k = 41) domain. A) Scatter plots of the individual effect sizes plotted as a function of the publication year with model predictions and 95% CI. B) Forest plots of the three-level meta-analytic model with effect sizes pooled by year with 95% CI. C) Forest plots of the cumulative effect sizes and 95% CI by year of publication.

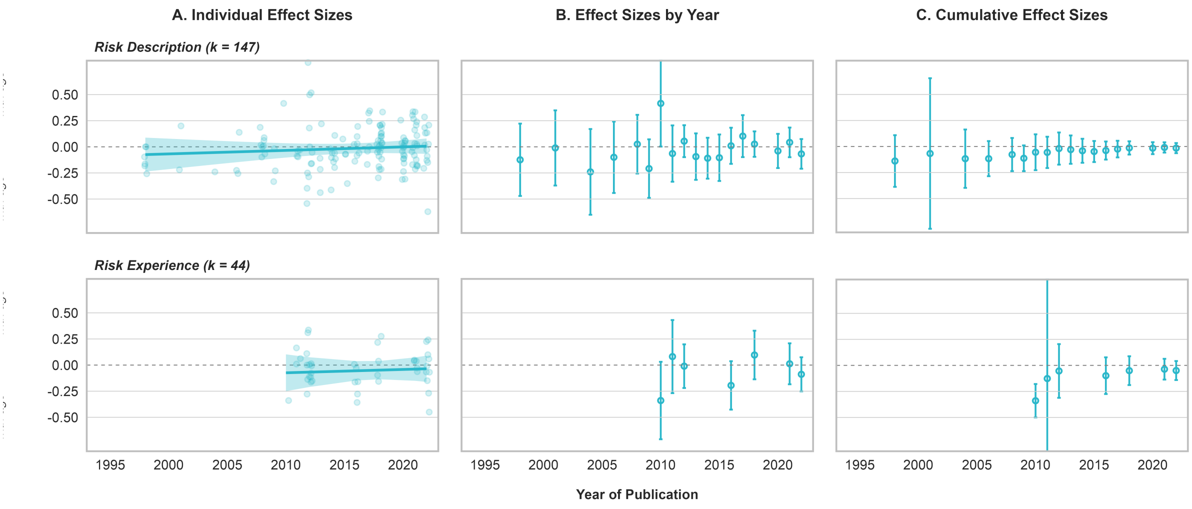

Figure S9

Meta-analytic results of the effect of age on risk taking in decisions from description (k = 147) or experience (k = 44). A) Scatter plots of the individual effect sizes plotted as a function of the publication year with model predictions and 95% CI. B) Forest plots of the three-level meta-analytic model with effect sizes pooled by year with 95% CI. C) Forest plots of the cumulative effect sizes and 95% CI.

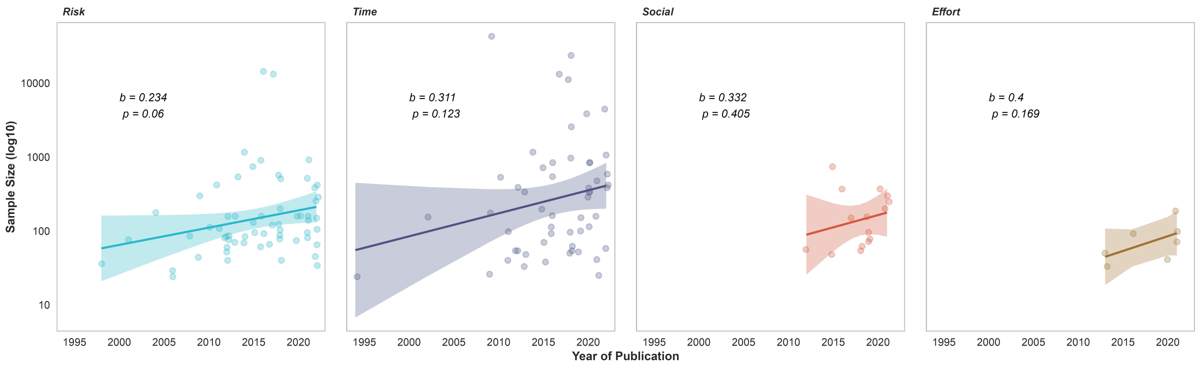

Figure S10

Association between the number of decades a study has been published as of 2022 (transformed into year of publication for plotting purposes) and study sample size for studies on age differences in risk (studies = 62), time (studies = 54), social (studies = 15), and effort (studies = 7) preferences. With model predictions and 95% CI. The beta value and the p-value are results of the linear regression with number of decades since publishing (year of publication - 2022) as a predictor.

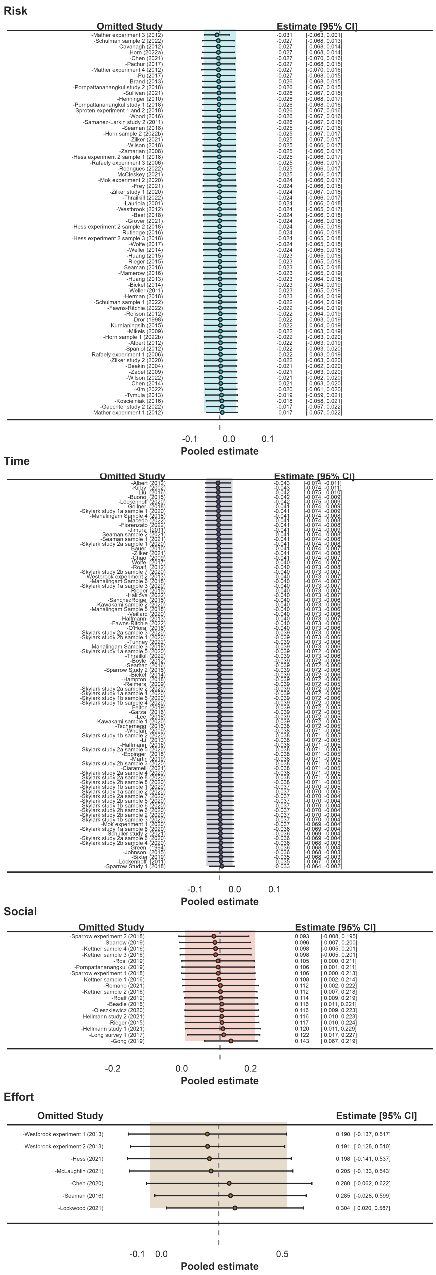

Figure S11

Leave-one-out sensitivity analysis for the three-level meta-analytic model on age differences in risk (k = 193), time (k = 125), social (k = 28), and effort (k = 23) preferences calculated by study. The highlighted section indicates the 95% CI, and the dotted line the mean of the pooled estimate from the three-level meta-analytic model.

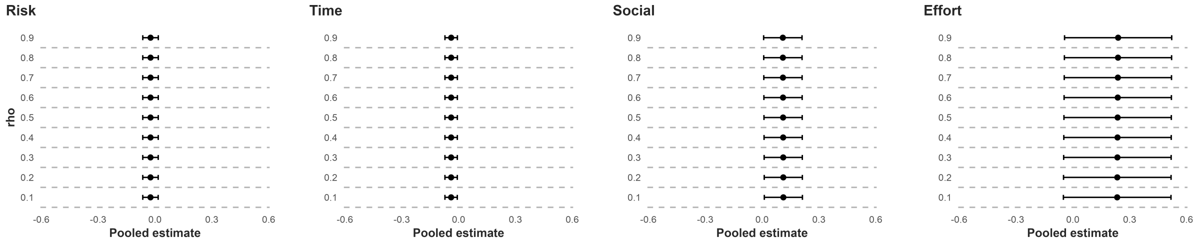

Figure S12

Plots of the pooled estimate for risk (k = 193), time (k = 125), social (k = 28), and effort (k = 23) preferences from the three-level meta-analytic model for different values of rho (i.e., correlation of sampling errors within studies).

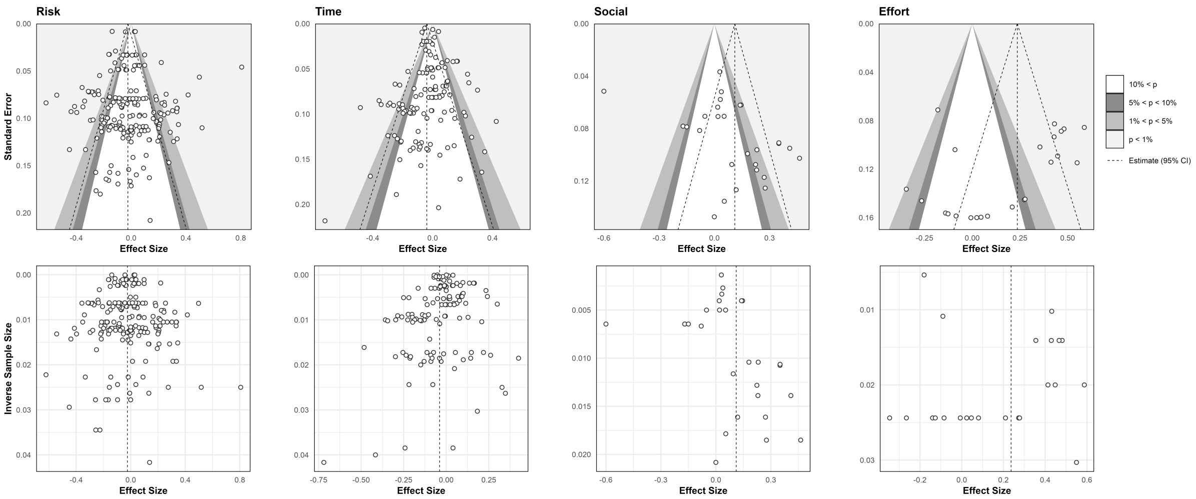

Figure S13

Funnel plots and contour-enhanced funnel plots of the effect sizes of primary studies on age differences in risk (k = 193), time (k = 125), social (k = 28), and effort (k = 23) preferences versus their standard error (upper) and inverse sample size (lower). The shaded regions of the contour-enhanced funnel plot indicate areas of statistical significance, and the white region represents non-statistical significance. The vertical line corresponds to the summary effect size estimate from the three-level meta-analytic model.

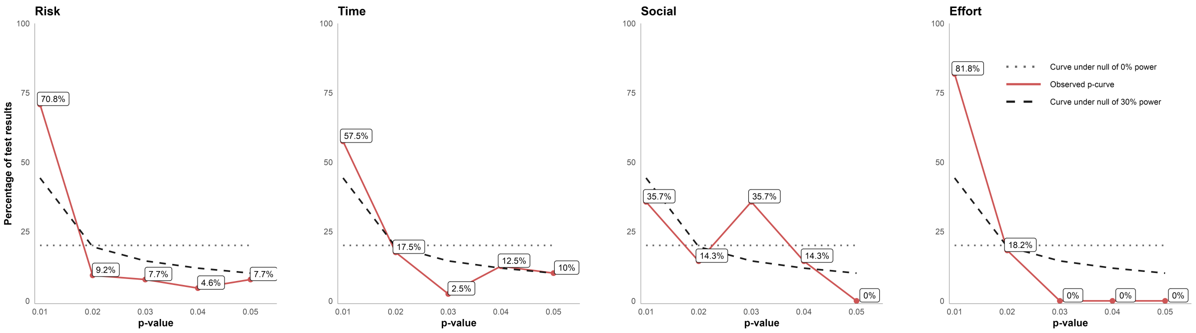

Figure S14

P-curve analysis with effect sizes of primary studies on age differences in risk (k = 193), time (k = 125), social (k = 28), and effort (k = 23) preference.

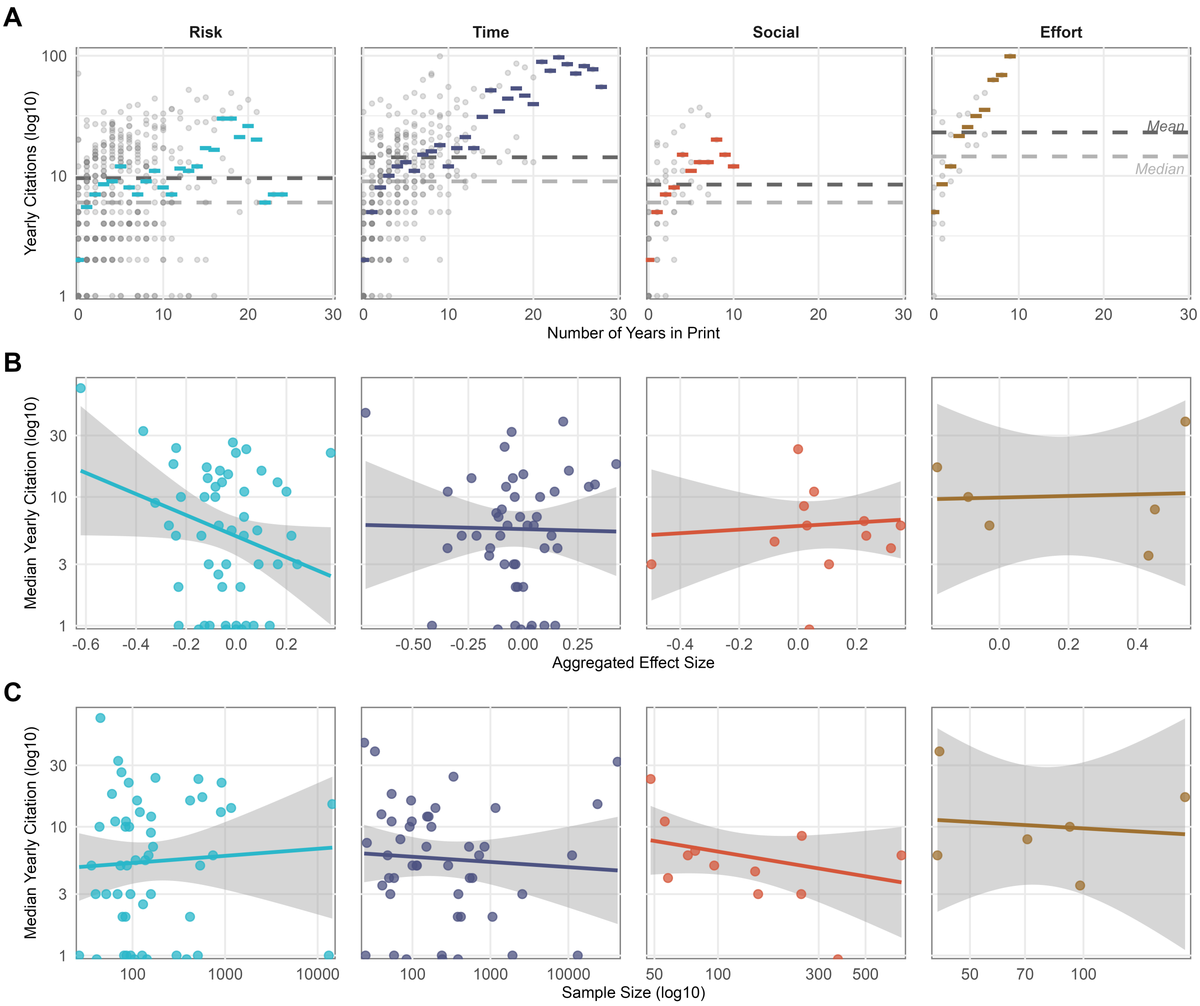

Figure S15

Association between citation count with number of years a publication has been available, the aggregated effect size and average sample size for publications on age differences in risk (publications = 54), time (publications = 48), social (publications = 12), and effort (publications = 6) preference. A) Yearly number of citations as a function of the number of years a publication has been in print. Thick colored dashes represent the median number of citations across all publications (grey dots) for each year. Dark and light grey dashed lines show the overall mean and median number of yearly citations, respectively. B) Scatter plots of the median yearly citation for each publication and its aggregate effect size, with a best fit line and 95%CI. C) Scatter plots of the median yearly citation for each publication and its mean sample size, with a best fit line and 95%CI.
